# Arabidopsis flippase ALA3 is required for adjustment of early subcellular trafficking in plant response to osmotic stress

**DOI:** 10.1093/jxb/erad234

**Published:** 2023-06-23

**Authors:** Vendula Pukyšová, Adrià Sans Sánchez, Jiří Rudolf, Tomasz Nodzyński, Marta Zwiewka

**Affiliations:** Mendel Centre for Plant Genomics and Proteomics, Central European Institute of Technology (CEITEC), Masaryk University (MU), Kamenice 5, CZ 625 00, Brno, Czech Republic; National Centre for Biomolecular Research, Faculty of Science, Masaryk University, Kamenice 5, 625 00 Brno, Czech Republic; Mendel Centre for Plant Genomics and Proteomics, Central European Institute of Technology (CEITEC), Masaryk University (MU), Kamenice 5, CZ 625 00, Brno, Czech Republic; National Centre for Biomolecular Research, Faculty of Science, Masaryk University, Kamenice 5, 625 00 Brno, Czech Republic; Mendel Centre for Plant Genomics and Proteomics, Central European Institute of Technology (CEITEC), Masaryk University (MU), Kamenice 5, CZ 625 00, Brno, Czech Republic; National Centre for Biomolecular Research, Faculty of Science, Masaryk University, Kamenice 5, 625 00 Brno, Czech Republic; Department of Experimental Biology, Faculty of Science, Masaryk University, Kamenice 5, 625 00 Brno, Czech Republic; Mendel Centre for Plant Genomics and Proteomics, Central European Institute of Technology (CEITEC), Masaryk University (MU), Kamenice 5, CZ 625 00, Brno, Czech Republic; Mendel Centre for Plant Genomics and Proteomics, Central European Institute of Technology (CEITEC), Masaryk University (MU), Kamenice 5, CZ 625 00, Brno, Czech Republic; Oklahoma State University, USA

**Keywords:** *Arabidopsis thaliana*, ARF, endocytosis, flippase, GEF, osmotic stress, protein trafficking, secretion

## Abstract

To compensate for their sessile lifestyle, plants developed several responses to exogenous changes. One of the previously investigated and not yet fully understood adaptations occurs at the level of early subcellular trafficking, which needs to be rapidly adjusted to maintain cellular homeostasis and membrane integrity under osmotic stress conditions. To form a vesicle, the membrane needs to be deformed, which is ensured by multiple factors, including the activity of specific membrane proteins, such as flippases from the family of P4-ATPases. The membrane pumps actively translocate phospholipids from the exoplasmic/luminal to the cytoplasmic membrane leaflet to generate curvature, which might be coupled with recruitment of proteins involved in vesicle formation at specific sites of the donor membrane. We show that lack of the AMINOPHOSPHOLIPID ATPASE3 (ALA3) flippase activity caused defects at the plasma membrane and *trans*-Golgi network, resulting in altered endocytosis and secretion, processes relying on vesicle formation and movement. The mentioned cellular defects were translated into decreased intracellular trafficking flexibility failing to adjust the root growth on osmotic stress-eliciting media. In conclusion, we show that ALA3 cooperates with ARF-GEF BIG5/BEN1 and ARF1A1C/BEX1 in a similar regulatory pathway to vesicle formation, and together they are important for plant adaptation to osmotic stress.

## Teaser

The interplay of P4-ATPase ALA3, ARF BEX1, and ARF-GEF BEN1 controls vesicle formation at the plasma membrane/*trans*-Golgi network and the subsequent vesicle trafficking important for the plant response to osmotic stress.

## Introduction

The plasma membrane (PM) is a frontier defining and protecting the cell. However, it serves not only as a mechanical barrier, but also as a platform where lipids, proteins, or polysaccharides are being exchanged between the intracellular compartments and apoplast in order to regulate cellular development and homeostasis (Liu *et al.*, 2015). Importantly, biological membranes play a fundamental role in compartmentalization of the cell interior. Intracellular compartments along the secretory and endocytic pathways are connected via membrane trafficking mediated by carrier vesicles, which ensures the delivery of building material and maintenance of membrane integrity (Shin *et al*., 2012).

To form a spherical vesicle, the membrane needs to be bent (Ungewickell and Hinrichsen, 2007), which is facilitated by several events encompassing the recruitment of coat complexes, scaffolding by the cytoskeleton, as well as membrane protein crowding, and changes in the lipid composition (McMahon and Boucrot 2015). Initiation of membrane curvature appears to be regulated by the small GTPases of the ADP-ribosylation factor family (ARF-GTPases) (Beck *et al.*, 2008). Their activity is controlled by switching between GTP/GDP-bound states, which is regulated by guanine nucleotide exchange factors (ARF-GEFs) and GTPase-activating proteins (GAPs), respectively. Whereas the inactive GDP-bound ARFs are cytosolic, their active GTP forms associate with the donor membranes, resulting in the recruitment of coat proteins that help to deform the membrane (McMahon and Mills, 2004). Additionally, a positive curvature towards the cytosolic side is promoted on the donor membrane by lipid asymmetry generated by the flippases from the family of P4-ATPases, which actively translocate specific phospholipids from the outer to the inner membrane leaflet (Sebastian *et al.*, 2012). Moreover, the local membrane curvature and subsequent vesicular budding depend on the shape of phospholipids and their spatiotemporal distribution. Flippases are therefore needed for the generation of a pool of specifically shaped phospholipids in the inner membrane leaflet (Janmey and Kinnunen, 2006). Newly formed vesicles pinch off from the donor membrane, thus initiating the transport of cargo proteins and lipids to their final destinations. The mature vesicles eventually fuse with the acceptor membrane in a tightly regulated fashion (Singh and Jurgens, 2018).

A considerable part of the published data about vesicular trafficking of plant PM proteins was generated in studies of PIN-FORMED (PIN) proteins, which mediate a directional transport of auxin facilitated by their asymmetric localization (Vieten *et al.*, 2007; Krecek *et al.*, 2009). PINs internalize via constitutive clathrin-mediated endocytosis (Kleine-Vehn *et al.*, 2011) and subsequently recycle to distinct PM subdomains (Langowski *et al.*, 2016) or are targeted to the vacuole for degradation (Geldner *et al.*, 2001; Dhonukshe *et al.*, 2007; Zwiewka *et al*., 2011; Nodzynski *et al.*, 2013). PIN trafficking is regulated by one of the most prominent ARF-GEFs named GNOM (Geldner *et al.*, 2003). In many cases, this GEF coordinates the intensive vesicular transport essential for PIN polarity switches during processes that require redirecting the auxin flux, such as shoot and root gravitropism (Rakusová *et al.*, 2011). This also shows why the rapidly recycling PINs can serve as a good tool to visualize and study subcellular dynamics.

Not only during tropisms, rapid delivery of cargos between the *trans*-Golgi network (TGN) and PM is one of the key mechanisms that the plant cell utilizes to adapt to stress conditions, such as soil salinity or drought (Zhu, 2016). Exogenous changes activate cellular mechanisms aiming to adjust endocytosis and exocytosis rates to fit current needs (Nakayama *et al.*, 2012). For example, exposing the cell to hypertonic conditions leads to its shrinking separation of the PM from the cell wall (Oparka, 1994; Zonia and Munnik, 2007) and enhanced internalization of PM proteins (Zwiewka *et al*., 2015). In contrast, a hypotonic environment increases intracellular turgor pressure, and the PM is pushed towards the cell wall (Zonia and Munnik, 2007), which results in enhanced exocytosis (Zwiewka *et al*., 2015). Several trafficking regulators were shown to play a role in plant adaptation to stress (Zhu *et al*., 2002; Drakakaki *et al.*, 2012; Hachez *et al.*, 2014). Recently, GNOM was identified as a key regulator of the cold stress response in plants. Interestingly, GNOM was shown to participate together with the AMINOPHOSPHOLIPID ATPASE3 (ALA3) flippase in the regulation of PIN recycling (Zhang *et al.*, 2020). ALA3 localizes to the Golgi, TGN, PM, and endosomes (Poulsen *et al.*, 2008; Zhang *et al.*, 2020), and its function is critical for both maintenance of primary cellular functions and the ability to cope with various growth conditions, such as soil composition or cold (McDowell *et al.*, 2013; Lopez-Marques, 2021). Consequently, *ala3* mutants exhibit pleiotropic phenotypes, including defective secretory vesicle formation in the columella root cap (Poulsen *et al.*, 2008), reduced growth of root and rosette (McDowell *et al.*, 2013), defects in gravitropic responses (Zhang *et al.*, 2020), abnormal trichome development (Zhang and Oppenheimer, 2009), fertility defects (Zhou *et al.*, 2020; Yang *et al.*, 2022), or sensitivity to pathogens (Underwood *et al*., 2017) and cold (McDowell *et al.*, 2013). Here, we further characterize the role of ALA3 in subcellular trafficking processes and its possible involvement in the regulation of plant response to osmotic stress. We analyzed two mutant alleles of *ALA3* (*are2* and *are3*), which were identified in a forward genetic screen described previously (Zhang *et al.*, 2020).

Our results indicate that the ALA3 flippase is necessary for proper TGN function, which is essential for the vesicle formation and subsequent secretory trafficking processes. Furthermore, we show that loss of *ALA3* results in a defective response to osmotic stress at the cellular and seedling level. Thus, we strengthen the previously reported importance of intracellular trafficking regulation during stress (Levine, 2002; Mazel *et al.*, 2004; Zwiewka *et al*., 2015) by providing more information about its role in the maintenance of subcellular dynamics.

## Materials and methods

### Plant material and growth conditions

Previously published *Arabidopsis thaliana* transgenic lines and mutants were used in this study: PIN1–green fluorescent protein (GFP) (Benkova *et al.*, 2003), PIN2–EGFP (Xu and Scheres, 2005), DR5rev::GFP (Friml *et al.*, 2003), SYP61–cyan fluorescent protein (CFP) (Robert *et al.*, 2008), SYP32–mCherry (Geldner *et al.*, 2009), CLC–GFP (Konopka *et al*., 2008), GFP:FABD (Ketelaar *et al.*, 2004), *are2* and *are3* (both in the PIN1–GFP background) (Zhang *et al.*, 2020), *ben1*/PIN2–GFP (Tanaka *et al.*, 2013), *bex1*/PIN1–GFP (Tanaka *et al.*, 2014), and *ala3-4*/PIN2–GFP (provided by Jiří Friml). To generate new lines for this study, PIN2–GFP, DR5rev::GFP, GFP:FABD, SYP61–CFP, SYP32–mCherry, CLC–GFP, *ben1*/PIN2–GFP, and *bex1*/PIN1–GFP were introduced into the *are*/PIN1–GFP mutants by genetic crossing.

Sterilized seeds were plated on Murashige and Skoog (Duchefa Biochemie) medium (MS+, where ‘+’ indicates the presence of sucrose) supplemented with 1% (w/v) sucrose and 0.8% (w/v) plant agar. After 2 d of stratification in the dark at 4 °C, seedlings were grown on vertically oriented plates under controlled conditions (16 h light/8 h dark cycles with 150 µmol m^–2^ s^–1^, at 21 °C) from 4 d to 6 d, depending on the assay. For phenotyping of the grown plants, seedlings were transferred to the soil (TS3 type, Klasmann Deilmann) and grown under controlled conditions (16 h light/8 h dark cycle at 21 °C).

### Measurements and statistical analysis

All measurements were performed using the ImageJ software package (National Institutes of Health, http://rsb.info.nih.gov/ij) (Schneider *et al*., 2012). For the signal intensity analyses, the mean gray value was measured in selected areas, depending on the assay. The Freehand line tool was used for measurements of the root length and brefeldin A (BFA) body area. The statistical analyses were done using the online freeware ASTATSA (https://astatsa.com/) or Student’s *t*-test.

### Seed coat ruthenium red staining assay

Staining of mature dry seeds was performed as described previously (McFarlane *et al*., 2014). The average thickness of the mucilage layer was measured using ImageJ software (Schneider *et al*., 2012).

### FM4-64 staining

Stock solution of 2 mM FM4-64 (Thermo Fischer Scientific) in water was used in all assays. To investigate the endocytosis changes, 5-day-old seedlings were incubated in MS+ containing 2 μM FM4-64 for 5 min on ice, washed three times at room temperature in MS+, mounted, and observed under a confocal microscope after 10 min. For endocytic rate measurements, the mean gray value of the entire cell was divided by the cell interior mean gray value. For the FM4-64 calibration, seedlings were incubated in liquid MS+ containing 2, 3, and 4 μM FM4-64 for 5 min on ice, washed three times at room temperature in MS+, mounted, and observed under a confocal microscope after 10 min. To quantify the staining of the PM under different dye concentrations, the mean gray value of PM regions was measured. For prolonged staining, 2 μM dye was used to stain the seedlings. After 20 min on ice, seedlings were washed three times at room temperature in MS+ and observed after 30 min.

### Immunodetection of ARF1 proteins in roots

For immunolocalization assay, 4-day-old seedlings were fixed with 4% paraformaldehyde (Merck) using vacuum infiltration for 60 min at room temperature. Automated whole-mount protein immunolocalization was performed as described previously (Sauer *et al.*, 2006). The anti-ARF1 rabbit antibody (Pimpl *et al.*, 2000) was used at 1:600 dilution. For the secondary antibody, we used CY3 anti-rabbit (Sigma-Aldrich) at a 1:600 dilution.

### Microscopy and fluorescence recovery after photobleaching (FRAP) data analysis

For the confocal laser-scanning microscopy work, LSM780 equipped with the C-Apochromat ×40/1.20 W objective (Inverted microscope Zeiss Axio Observer Z1) or LSM880 equipped with the C-Apochromat ×63/1.20 W (Inverted microscope Zeiss Axio Observer 7) was used. Various confocal settings were used to record the emission of the used fluorophores. The following values were taken from image acquisition settings of the Zeiss confocal software and represent the best compromise between the microscope hardware and fluorophore spectral properties that still allows good image acquisition and signal detection of a particular fluorophore. GFP (excitation 488 nm, emission 546 nm, detection 493–598 nm), CFP (excitation 458 nm, emission 516 nm, detection 463–568 nm), mCherry (excitation 561 nm, emission 643 nm, detection 568–718 nm), FM4-64 (excitation 514 nm, emission 675 nm, detection 592–758 nm), and CY3-conjugated secondary antibody (excitation 555 nm, emission 573 nm, detection 560–800 nm).

For the FRAP experiments, 5-day-old seedlings were used. During analyses of SYP32–mCherry, SYP61–CFP, and CLC–GFP, the FRAP mode of the LSM780 ZEN software was set up for two pre-bleach images followed by bleaching (six iterations, 100% argon-ion laser transmission, 1.58 µs pixel dwell time) and 40 post-bleach scans. The rectangular area of the bleached region [region of interest (ROI)] was 1000 µm^2^. Images of 512 × 512 pixels were acquired unidirectionally within 1.58 s/frame, and recovery of the fluorescence was recorded within 119 s. For the PIN2–GFP analysis, FRAP mode of the LSM880 ZEN software was set up for two pre-bleach images followed by bleaching (six iterations, 100% argon-ion laser transmission, 4.10 µs pixel dwell time) and 40 post-bleach scans. The circular ROI area was 19.6 µm^2^. Images of 512 × 512 pixels were acquired unidirectionally within 1.26 s/frame, and recovery of the fluorescence was recorded within 649 s. To normalize the acquired data and frame them in a range of values from 0 to 1, we normalized to the pre-bleach intensity. The pre-bleach value thus represents the intensity which the region possessed before the bleaching started. This normalization aimed to correct for the putatively unequal PIN expression in the lines. The ImageJ plugin ‘FRAP analysis’ was utilized as described previously (Phair *et al*., 2004). To fully normalize measurements in comparison with the curve fitting that assumes that the bleaching point equals 0, a further normalization step is required using the full normalization equation Full norm(t)=[FRAP(t)–FRAPbleach]/[FRAP(pre)–FRAPbleach], where FRAP(t) is the intensity of the reference region at time point t, FRAP(pre) is the mean intensity of the pre-bleach FRAP region, and FRAPbleach is the intensity of the FRAP region at the time of bleaching.

When modeling a dataset, we used the FIJI’s Curve Fitting feature. The normalized recovery curve was constructed using the post-bleach section after selecting the ‘Exponential Recovery’ curve. We used the following parameters to fit the curve: if we achieved complete recovery, a+c=1. Thus, the immobile fraction, f_immo = 1–a–c, or f_mobile=a+c (a is a slowly recovering fraction, c is a rapid diffusion fraction, b is the recovery rate, and when assuming uncoupled diffusion and binding, this corresponds to k_off=b). Our goal was to have 20–30 replicates per line, which increased the reliability of the mean mobile fraction values. Based on the standard error calculations of replicate experiments, we indicated that our analysis was variable, but the standard deviation also provided this information. In some cases, drift was corrected using FIJI’s Registration Plug-in (http://fiji.sc/StackReg).

For the ruthenium red staining assay, the transmitted light microscope Zeiss Axioscope.A1 (objective ×20/0.5) equipped with the Axiocam 105 camera and the software Visitrone Visiview was used.

### Brefeldin A treatment

Stock solution of 50 mM BFA (Sigma-Aldrich) in DMSO was used. In all assays, 5-day-old seedlings were incubated in liquid MS+ containing BFA. Subcellular responses were analyzed in seedlings treated with 25 µM BFA and imaged after 1 h. For the BFA washout experiment, seedlings were treated with 25 µM BFA, then transferred to liquid MS+ to wash out the excess dye and imaged after 30 min. For the BFA sensitivity assay, seedlings were treated with 25 µM BFA and imaged after 10, 15, and 20 min.

### Osmotic stress recovery assay

In the plate-based osmotic stress recovery assay, 5-day-old seedlings were transferred from the germination medium to fresh MS+ control plates with or without 200 mM mannitol. To be able to measure the root growth response, the positions of the root tips were marked. After 2 d of growth, plates were scanned and the distances from the marks to the root tips were measured. In the next step, seedlings were transferred to fresh MS+ plates and root tips were marked. Plates were scanned after 2 d of recovery and the distances from the marks to the root tips were measured using the ImageJ software.

For the cellular response to osmotic stress and recovery, root epidermal cells of 5-day-old seedlings grown on MS+ plates were imaged using a confocal microscope. Next, seedlings were transferred to MS+ plates containing 200 mM mannitol and images were acquired after 3 d. For the recovery, seedlings were placed back on the MS+ medium plates and observed after 1 d and 3 d. The mean gray value of the signal was measured in single planes of all the acquired images (12–15 images/time point) using the ImageJ software.

### Quantitative reverse transcription–PCR (RT–qPCR)

Five-day-old *A. thaliana* Col-0 seedlings were immersed in liquid MS+ supplemented with 200 mM mannitol. Seedlings were frozen in liquid nitrogen at time 0 and after 3, 6, 9, 12, and 24 h of mannitol treatment. Total RNA was extracted with a TRIzol Reagent (Thermo Fisher Scientific) as described in the TRIzol User Guide, and cDNA was synthesized using an iScript cDNA kit (Bio-Rad).

For ALA3 (AT1G59820) quantification, primers ALA3-F (AGCCAAATCTGCATTACGAGACC) and ALA3-R (TCTCGATGAGTTCTGCCACCTC) were acquired from Integrated DNA Technologies (IDT). The product amplicon has 62 nucleotides and is intron spanning (exon 19–exon 20) in the only one AT1G59820.1 gene model annotated for ALA3 in TAIR10. Therefore, alternative splice variants were not considered. The cDNA and genomic DNA specificity was checked in primer designing software QuantPrime (Arvidsson *et al.*, 2008). UBIQUITIN-CONJUGATING ENZYME 10 (UBC10; AT5G53300) was used as a reference gene (Czechowski *et al.*, 2005). Primers UBC10-F (CAAGGTGCTGCTATCG) and UBC10-R (ATCTCGGGCACCAAAGG) were acquired from Sigma-Aldrich. The product amplicon has 69 nucleotides and involves exon 4 and exon 5 of AT5G53300.1 and corresponding exons of all other splice variants annotated in TAIR10. ELONGATION FACTOR 1ALFA (EF1A; AT5G60390) was used as a second reference gene. Primers EF1-F (TGAGCACGCTCTTCTTGCTTTCA) and EF1-R (GTGGTGGCATCCATCTTGTTACA) were acquired from Sigma-Aldrich. The product amplicon has 75 nucleotides and involves exon 2 and exon 3 of AT5G60390.1 and corresponding exons of all other splice variants annotated in TAIR10. All oligos were purified by standard desalting only and shipped in the lyophilized state.

The samples were run as stated: denaturation at 95 °C 5 min; 45 cycles 95 °C 10 s, 60 °C 20 s, 72 °C 20 s; hold 72 °C 1 min; melt curve ramp 55–90 °C with 0.5 °C increment, 5 s for each step. Melt curves of sample products were checked in every run. Before the run, samples were held at 4 °C. The 20 µl qPCR mixture was composed of 10 µl of LightCycler 480 SYBR Green I Master (Roche, 2× dilution), 1 µl of forward primer solution (10 µM), 1 µl of reverse primer solution (10 µM), 4 µl of PCR-grade twice-sterilized MQ water, and 4 µl of 10× diluted reverse transcription mix (thus 20 ng of DNA). The master mix was handled based on provider instructions. The mixture was manually pipetted into tubes suitable for the 72-well Rotor gene Q, manufactured by Qiagen GmbH. Each cDNA sample was performed in triplicate. Primer pair efficiencies were estimated in 10× dilution series covering five orders of magnitude.

Cycle threshold (Ct) values higher than 35 were not accepted since this is near the position of the no-RT (NRT) control. All efficiencies were present in between the generally accepted range 0.9–1.0, with *R*^2^ >0.99. Ct values were determined using Rotor-Gene Q Series Software version 2.3.5 by inbuilt quantification, utilizing a graph of normalized fluorescence with correction of average background of each sample prior to amplification (‘dynamic tube’) and noise slope correction. The threshold was set to 0.01. The Ct values of qPCR technical triplicates were averaged for each gene and an average value from the non-treated control was subtracted. Resulting numbers were negatively exponentially transformed with the basis of 2. Then, the values of the gene of interest were divided by the geometrical mean of values corresponding to reference genes. This step provided us with the relative expression of our gene of interest. The graph shows the ratio of mannitol-treated and MS-treated samples.

The statistical analysis was peformed in RStudio version 2022.7.2.576 (R Studio Team, 2020) using R version 4.2.1 (R Core Team, 2022). A linear mixed model was used since it represents a better-fitting alternative for conventional analysis of variance for qPCR data (Steibel *et al.*, 2009). Aiming to distinguish between technical and biological sources of variance, we built a model with time and treatment as fixed factors and experimental replication as a random component (with a random intercept). The contrasts were evaluated only between control and mannitol-treated samples at each time point (Kenward–Roger method for degrees of freedom estimate, Tukey method for *P*-value adjustment). The analysis utilized packages: openxlsx (Schauberger and Walker 2022), lme4 (Bates *et al.*, 2015), and emmeans (Lenth, 2023).

## Results

### Flippase ALA3 is necessary for plasma membrane PIN protein abundance and lateral diffusion within a lipid bilayer

**Fig. 1.**
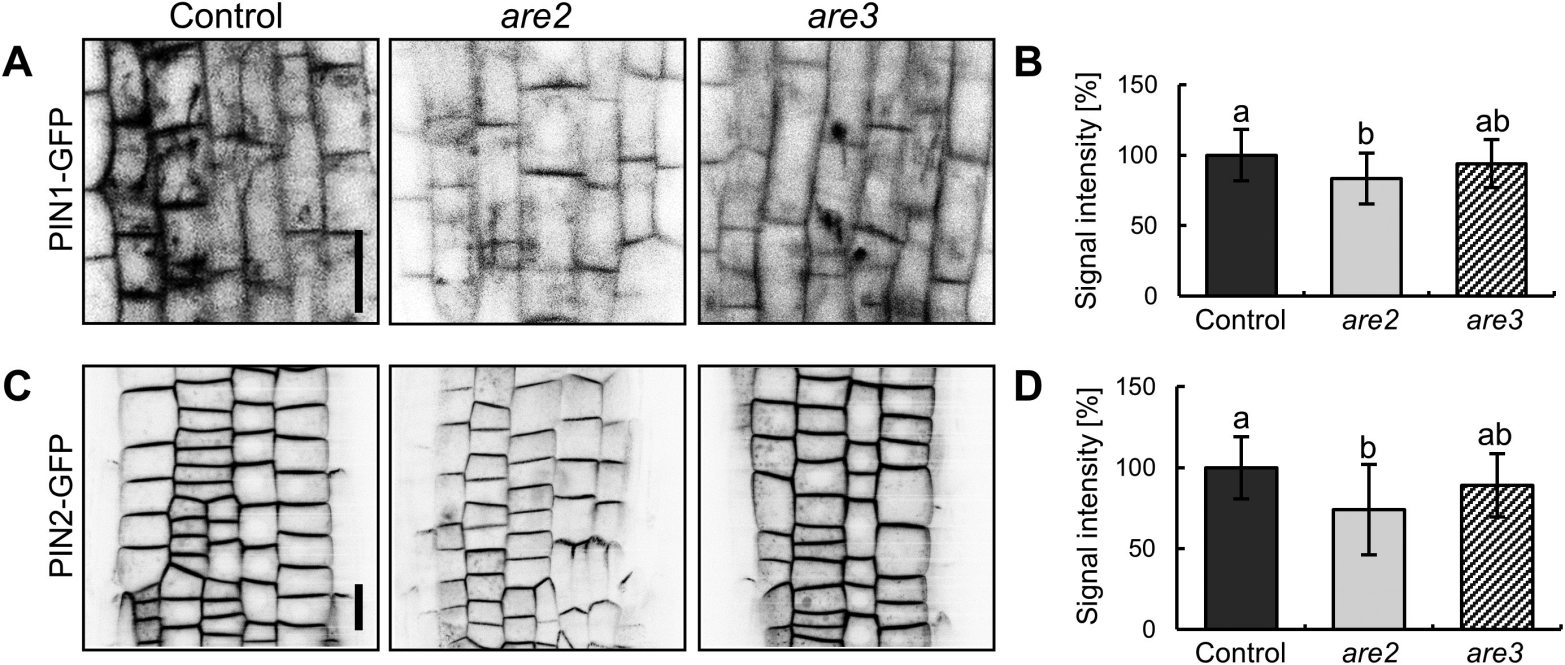
ALA3 is necessary for the PM PIN protein abundance. (A) Representative images of epidermal root cells in the control, *are2*, and a*re3* seedlings, all in the PIN1–GFP background. Scale bar=10 µm. (B) Quantification of the PIN1–GFP fluorescent signal. Error bars indicate the SD, and average results from three independent experiments (21 seedlings/line) are presented. (C) Representative images of epidermal root cells in the control, *are2*, and *are3* seedlings, all in the PIN2–GFP background. Scale bar=10 µm. (D) Quantification of the PIN2–GFP fluorescent signal. Error bars indicate the SD, and average results from three independent experiments (23–25 seedlings) are presented. The columns in (B) and (D) sharing the same letters are not significantly different from each other (one-way ANOVA with Tukey post-hoc test, *P*<0.01).

As mentioned above, a lipid bilayer is an environment in which membrane proteins reside and perform their function. They are delivered and removed from the PM by vesicular transport. We were aware that any subcellular trafficking defect can be better visualized when testing proteins that rapidly cycle between the PM and internal compartments (Zwiewka and Friml, 2012; Nodzynski *et al.*, 2013). Therefore, we investigated the subcellular localization of intensively recycling PIN efflux carriers in the *are* flippase mutants that we isolated and introduced into the PIN1–GFP genetic background already in our previously published contribution (Zhang *et al.*, 2020). To test the PM abundance of PIN1 in the *are* seedling roots, we performed live confocal imaging and measured the intensity of the PIN1–GFP signal. Additionally, we quantified the PM levels of PIN2 in the *are*/PIN2–GFP crosses in the same way. Whereas the *are3* seedlings rather resembled the phenotype of the control, a significant reduction of both PIN1 and PIN2–GFP signals was observed in *are2* (Fig. 1A–D). It was proposed that within the PM, PINs are recruited into the non-mobile clusters, which significantly decreases their lateral diffusion and supports the maintenance of their polar PM localization (Kleine-Vehn *et al.*, 2011). The PM cargo diffusion can be visualized best by utilizing the PIN2 expressed in root epidermal cells. Thus, we performed FRAP on a 5 µm long ROI in the PM, followed by a 10 min long semi-quantitative monitoring of the fluorescence recovery (Supplementary Fig. S1A–C). The PIN2–GFP signal recovery was clearly faster in both mutants, but the effect was statistically significant for the *are2* allele. To make sure that the difference in fluorescent signal recovery is directly related to lack of ALA3, we subjected the *ala3-4* knockout line (Poulsen *et al.*, 2008) crossed with PIN2–GFP to similar FRAP analysis. As for *are2*, we could observe a significantly faster GFP signal recovery (Supplementary Fig. S1D–F). Thus, our results indicate that lack of ALA3 flippase affects the PM PIN protein abundance and speeds up the PIN2 PM diffusion rate.

Could the above-mentioned defects translate into macroscopic phenotypes? For PIN2 harboring mutations in Cys39 and Cys560 in minor loops connecting the α-helices, increased clustering resulting in a less wavy root was reported. Similarly, less root meandering was seen for the *pin2* mutant (Retzer *et al.*, 2017). Therefore, we wondered if similar phenotypes could be observed in *are* mutants. We did not notice root waving changes but, interestingly, we observed that the *are* mutant phenotype is reminiscent of seedlings grown on medium supplemented with auxin. The primary roots of mutant seedlings are significantly shorter than those of the control (Supplementary Fig. S2A, B) and we measured a significantly higher density of root hairs in both *are2* and *are3* (Supplementary Fig. S2C, D). Both of those phenotypes can be caused by increased auxin levels in the root. The distribution of auxin in plants can be monitored by utilizing synthetic auxin-responsive promoters, such as DR5 (Ulmasov *et al.*, 1997). Here, we integrated the *DR5rev::GFP* reporter into the mutant background and observed a significant increase of the DR5 signal in the *are* root caps versus the control (Supplementary Fig. S2E, F), confirming that the auxin accumulation is enhanced in the mutant seedlings. Since the intracellular recycling of PINs is so dynamic, it is worth mentioning that many trafficking defects will result in their mislocalization, which will in turn result in some auxin-related phenotypes. However, not all defects need to be entirely PIN specific.

### Flippase ALA3 is involved in the early steps of endocytosis and secretion but not in intracellular vesicular dynamics

**Fig. 2.**
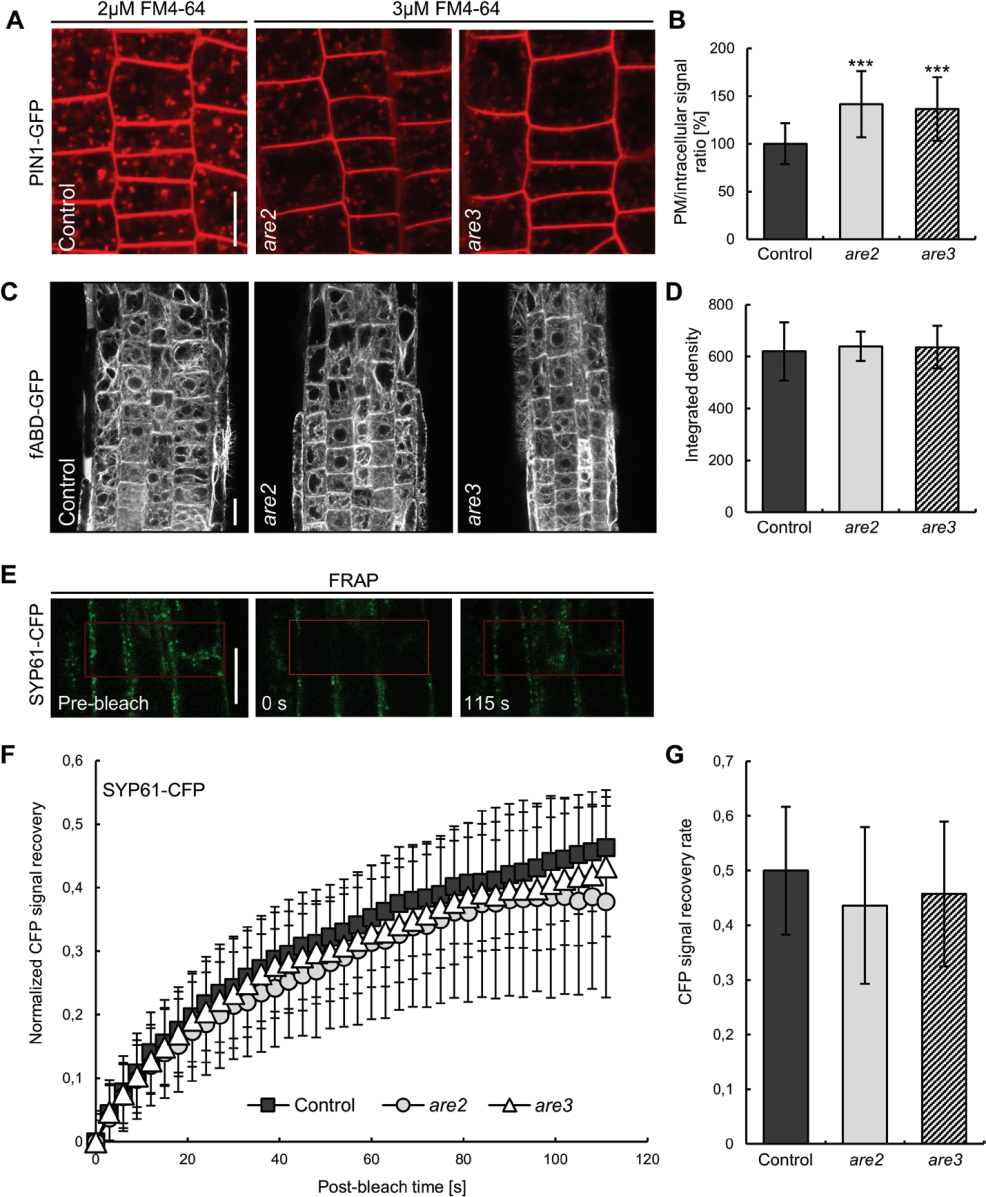
ALA3 is involved in the early steps of endocytosis but not in the intracellular endosomal transit dynamics. (A) Confocal images of epidermal root cells of PIN1–GFP control and its crosses with *are2* and *are3* after brief FM4-64 staining. Scale bar=20 µm. (B) Quantification of the FM4-64 internalization (PM to intracellular signal ratio). Error bars indicate the SD (>40 cells from two independent experiments). Asterisks mark significant differences (Student’s *t*-test, ***P<0.001). (C) Intracellular localization of the actin filament marker fABD–GFP in Col-0 control and the mutant background crosses with *are2* and *are3*. Scale bar=20 µm. (D) Quantification of the integrated density of actin filaments. Error bars indicate the SD. (E) FRAP experiment—representative images of the TGN-localized SYP61–CFP marker acquired at the indicated time points of the FRAP experiment. (F) Representative normalized recovery curve after FRAP analysis of SYP61 in Col-0 (squares) and the mutant background crosses with *are2* (circles) and *are3* (triangles). Error bars indicate the SD. (G) Mean recovery rate expressed as percentage of CFP signal in SYP61–CFP and its crosses with *are2* and *are3* from 30 individual FRAP experiments. Error bars indicate the SD.

Live confocal imaging of FM4-64 uptake is a valuable method that enables evaluation of general endocytosis rates and is not delimited to a specific protein cargo. In plants, the dye initially intercalates into the outer leaflet of lipid bilayers and emits a strong fluorescence in such a hydrophobic environment. Moreover, positively charged heads of the dye moiety prevent its movement between the two PM leaflets (Jelinkova *et al.*, 2010). Subsequently, FM4-64 internalizes together with the membrane and, over time, it labels all the compartments along the endocytic pathway to finally stain the tonoplast (Kutsuna *et al.*, 2003; Jelinkova *et al.*, 2010). Taking advantage of the endocytic tracer, we sought to explain altered PIN1 and PIN2 levels at the *are* mutant PM (Fig. 1A–D). We wondered whether endocytosis rates were generally affected, and if the PIN PM abundance changes were just one facet of a more extensive trafficking defect in *are* mutants. To visualize the very first endocytosis steps involving formation of vesicles at the PM and their trafficking towards the early endosomes, we utilized FM4-64 to stain the membranes of the PIN1–GFP control and its crosses with *are* plants. Our initial analysis revealed that the PM of *are* seedlings was labeled less effectively compared with the control after application of 2 µM FM4-64 for 5 min and a subsequent 10 min washout. To enable a more accurate endocytic rate evaluation, we equalized the staining of both control and mutant PMs. In our experimental conditions, treating the mutant seedlings with 3 µM FM4-64 resulted in the same staining intensity as when applying the standard 2 µM concentration of dye to the control epidermal cells (Supplementary Fig. S3A, B). With adjusted dye concentrations, we could proceed to test the FM4-64 internalization in all lines, and our results showed that the PM to intracellular signal ratio is significantly higher in the flippase mutants, indicating a decreased endocytosis (Fig. 2A, B).

Since cytoskeleton components, such as actin filaments and microtubules, participate in vesicle transit inside the cytoplasm (Bhaskar *et al*., 2007), we decided to track localization of the fABD–GFP marker (van der Honing *et al*., 2011) consisting of GFP fused to the second actin-binding domain of Arabidopsis fimbrin1 (Fig. 2C, D). However, we did not observe any striking change in the pattern of actin filaments, which led us to associate altered trafficking rather with fewer events of vesicle formation than with their slower transit through the cytoplasm. To corroborate those results, we utilized FRAP to investigate the cytoskeleton-dependent early endosome movement dynamics in the cytoplasm. By a semi-quantitative approach, we addressed the migration of SYP61–CFP, a TGN/early endosome (EE)-localized SNARE playing a key role in membrane trafficking (Jahn and Scheller 2006) (Fig. 2E–G). Assuming that fluorescent protein bleaching does not affect the SYP61 dynamics, it is reasonable to relate the recovery of the CFP signal to the speed of intracellular endosome transit from the bleached to the unbleached region. Our results did not show any significant difference in the signal recovery rate after photobleaching. Similarly, when we analyzed another SNARE, a Golgi-localized SYP32 tagged with mCherry, that we introduced into the *are* mutant background, we did not observe any signal recovery differences (Supplementary Fig. S4A–C). Moreover, the GFP signal recovery after photobleaching was also not affected in mutant crosses with clathrin light chain (CLC), one of the components of the clathrin-coated vesicles (Supplementary Fig. S4D–F). Thus, our results suggest that the ALA3 genetic lesion does not affect general cytoplasmic transit of endosomes or organelles reliant on cytoskeleton function.

**Fig. 3.**
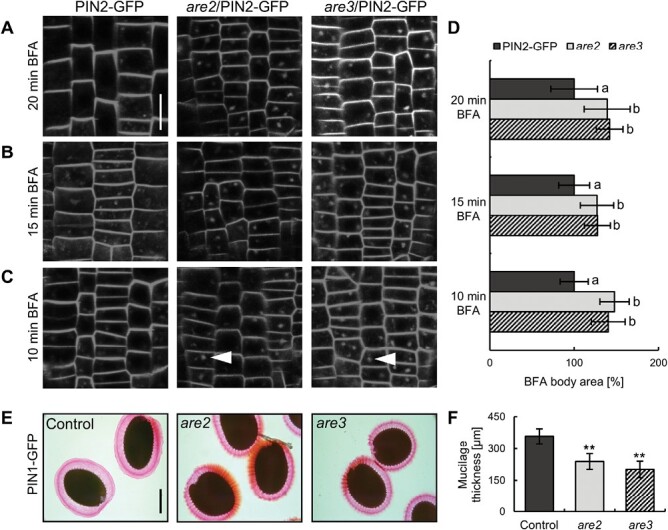
ALA3 is involved in TGN function and the secretory processes. (A–C) Visualization of PIN2 accumulation in BFA bodies in PIN2–GFP and its crosses with *are2* and *are3* after application of 25 µM BFA for 20 (A), 15 (B), and 10 min (C). Scale bar=10 µm. (D) Quantification of BFA body area. Error bars indicate the SD (>30 BFA bodies). Columns sharing the same letters are not significantly different from each other (one-way ANOVA with Tukey post-hoc test, *P*<0.01). (E) Images of ruthenium red-stained seed coat mucilage of the PIN1–GFP control and its crosses with *are2* and *are3* lines. Scale bar=500 µm. (F) Quantification of the thickness of the extruded mucilage layer in seeds shown above. Error bars indicate the SD (>30 seeds). Asterisks mark significant differences (Student’s *t*-test, **P<0.01).

Bearing in mind the defects in endocytosis, we decided to investigate exocytosis and gain more detailed picture of possible trafficking pathways alterations in *are* mutants. For this purpose, we utilized BFA, a well-studied chemical inhibitor of protein recycling from the TGN/EE to the PM. On the cellular level, its effect can be observed as the formation of so-called BFA bodies consisting of aggregated EE, TGN, and partially Golgi stacks (Nebenfuhr *et al*., 2002). Washing the inhibitor out of the cell restores the transit of aggregated cargos back to the PM, and such BFA body disappearance can be related to the exocytosis rates (Geldner *et al.*, 2001; Kleine-Vehn *et al*., 2008). Recently, it was shown that application of BFA results in increased size and number of BFA bodies per cell in *ala3* mutants (Zhang *et al.*, 2020). In our study, application of 25 µM BFA for 1 h followed by a 30 min MS+ washout resulted in a significantly larger area of PIN1-labeled BFA bodies in *are* mutants crossed with PIN1–GFP (Supplementary Fig. S5A, B). To further investigate the *are* mutant sensitivity to BFA and to address the trafficking of PIN2 as another PM cargo, we systematically analyzed different exposure times to the drug by incubating seedlings in MS+ medium containing 25 µM BFA, decreasing the length of treatment from 1 h to 20/15/10 min (Fig. 3A–C) and comparing the area of PIN2 aggregations in BFA bodies between the PIN2–GFP control and its crosses with *are* mutants within each treatment (Fig. 3D). In all experimental conditions, even after 10 min treatment, significantly larger PIN2-labeled BFA bodies were observed in *are* seedlings, indicating strong oversensitivity of those mutants to BFA. Thus, the retention of intracellular accumulations, that we observed in *are* mutants, might be rather reflecting how slowly the TGN and endosomes disaggregate from BFA bodies, and it therefore does not very precisely relate to the speed of exocytosis. Keeping this in mind, we came up with an alternative experiment to analyze the general exocytosis rates and intracellular material secretion during seed imbibition. The differentiated Arabidopsis secretory seed coat cells represent a useful model system for studying these processes. Development of the seed coat involves a tightly regulated series of events, including biosynthesis and secretion of a pectin-rich gelatinous layer called mucilage (Western *et al*., 2000). Mucilage components are produced within the Golgi stacks, from where they are delivered in large amounts to specific cell wall domains in the form of secretory vesicles (Young *et al.*, 2008). Immersing seeds in an aqueous solution triggers imbibition and leads to extrusion of hydrated mucilage, which can be visualized as a pink-stained layer surrounding the seeds upon staining with ruthenium red dye (Western *et al*., 2000). A reduced mucilage staining was visible in *are* mutants (Fig. 3E, F), which may be a result of defects in secretion. To investigate what could be the cause of secretory problems, we focused on the Golgi/TGN, which serves as a major sorting hub where secretory and endocytic routes cross (Dettmer *et al.*, 2006; Viotti *et al.*, 2010). For this purpose, we utilized the ARF1 protein, which resides in Golgi/TGN compartments and plays a crucial role in BFA-sensitive vesicular trafficking (Naramoto *et al.*, 2010; Robinson *et al.*, 2011). Immunostaining using *anti-*ARF1 antibody showed that in both *are2* and *are3*, the protein localizes in aggregates, indicating morphological changes in TGN compartments (Supplementary Fig. S5C). Additionally, we stained the seedlings with FM4-64 for 20 min, to label the TGN/EE. Afterwards, we washed out the excess dye and, after 30 min, we detected FM-labeled accumulations in the early compartments that probably correspond to the TGN (Supplementary Fig. S5D) in mutant seedlings. These experiments, as well as the BFA oversensitivity (Fig. 3A–D), point to a TGN disruption in *are* mutants. Congruously, ALA3 flippase was proposed to participate in formation of transport vesicles from the TGN (Poulsen *et al.*, 2008; Underwood *et al*., 2017; Zhang *et al.*, 2020).

From our data, we can conclude that lack of ALA3 negatively influences endocytosis and exocytosis, but it does not affect the speed of the intracellular cargo transit after it is dispatched from the TGN or PM. It seems logical to infer that the *ALA3* mutation slows down the steps of vesicle formation on the donor membranes where the flippase is natively functioning.

### Flippase ALA3, ARF-GEF BEN1, and ARF BEX1 are necessary for a proper TGN function

As mentioned above, the cooperation of multiple molecular players is required for the formation of transport vesicles at the donor membranes. We have so far assumed that some of the steps might be impaired in *are* mutants. It was shown that ARFs and ARF-GEFs regulate vesicle formation by associating with the membrane and triggering the recruitment of the coating components (Luschnig and Vert, 2014). Furthermore, genetic and physical interaction studies revealed that ALA3 flippase functions together with some ARF-GEFs in controlling PIN polarity, trafficking, and auxin-mediated plant development (Zhang *et al.*, 2020). To shed light on the role of ALA3 in the above-mentioned processes, we introduced into our study ARF1A1C/BEX1 and ARF-GEF BIG5/BEN1. Both proteins localize to the TGN/EE and Golgi, and act synergistically in regulation of early endosomal trafficking (Tanaka *et al.*, 2009). Since *bex1* and *ben1* mutant phenotypes were previously characterized, we could utilize them as additional tools to understand the trafficking processes in the flippase mutants. Whereas *bex1* is hypersensitive to BFA, *ben1* does not show any intracellular agglomerations after BFA treatment (Tanaka *et al.*, 2014). We generated crosses of *are2* with *bex1* and *ben1*, all in the PIN1–GFP genetic background, and tested BFA sensitivity in the double mutants.

**Fig. 4.**
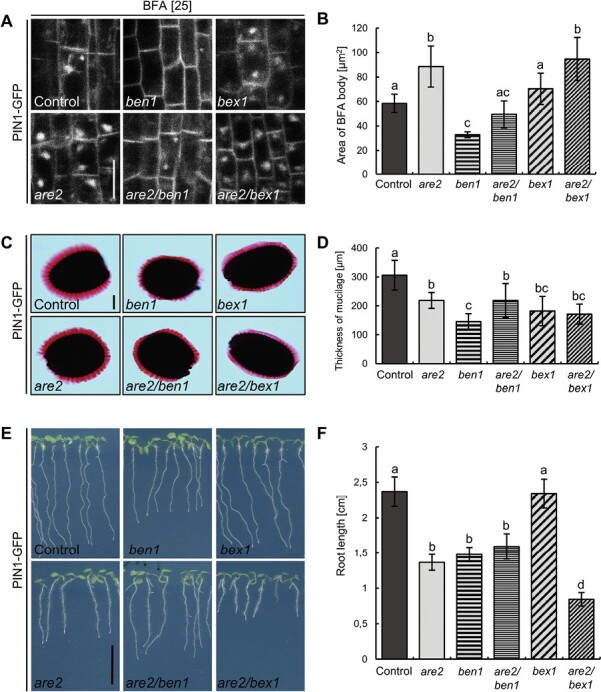
ALA3, BEN1, and BEX1 are necessary for proper TGN function. (A) PIN1 accumulation in BFA bodies after incubation for 1 h and subsequent 30 min washout of the drug with MS+ medium for the control, *are2*, *ben1*, *bex1*, and their double mutants, all in the PIN1–GFP genetic background. Scale bar=10 µm. (B) Area quantification of the PIN1-containing BFA bodies. Error bars indicate the SD (>30 BFA bodies). (C) Images of ruthenium red-stained seed coat mucilage of the control, *are2*, *ben1*, *bex1*, and their double mutants, all in the PIN1–GFP genetic background. Scale bar=500 µm. (D) Quantification of the thickness of the extruded mucilage layer in the seeds shown above. Error bars indicate the SD (>30 seeds). (E) Representative images of 6-day-old seedlings grown on MS+ medium. (F) Root length measurements. Error bars indicate the SD (>25 seedlings). In all graphs, columns sharing the same letters are not significantly different from each other (one-way ANOVA with Tukey post-hoc test, *P*<0.01).

Treatment with 25 µm BFA for 1 h and a subsequent 30 min long washout of the drug was followed by measuring the area of the remaining BFA bodies visualized by the presence of PIN1–GFP (Fig. 4A, B). A higher accumulation of PIN1 was observed in the *are2* mutant when compared with the control. While the BFA body area was not changed in *bex1*, we could observe an enhanced *are2* intracellular phenotype in *are2/bex1*, indicating that the *are2* mutation is epistatic over *bex1*. For *ben1*, we noticed a decreased BFA body area, which corresponds to the reported mutant resistance to this inhibitor. Even though the BFA body area in *are2/ben1* was increased in comparison with the *ben1* single mutant, it did not reach the level of the *are2* phenotype as it did in *are2/bex1*. However, *are2/ben1* exhibited a partially restored phenotype.

We showed above by measuring the layer of the extruded mucilage that secretion is less effective in the *are* mutants (Fig. 3E, F). Here, a similar analysis revealed that when compared with the control, all tested single and double mutants show defects in secretion (Fig. 4C, D). In addition, whereas the *are2/ben1* double mutant resembled the *are2* phenotype and, when compared with *ben1*, the mucilage was significantly thicker, we did not observe a significant difference between *are2/bex1* and the *are2* or *bex1* single mutants. Because of the additive effect of *are2* mutation after BFA treatment in *are2/bex1*, we think that ALA3 and BEX1 might function in the same regulatory pathway.

Interestingly, we did not see the above-described secretory defects reflected in the seedling phenotypes (Fig. 4E, F). Measurement of the primary root length of 6-day-old seedlings revealed a similar decrease in the *are2*, *ben1*, and *are2/ben1* seedlings. Whereas *bex1* root length resembled that of the control, a significant decrease was observed in *are2/bex1*. These differences were even more evident in grown plants (Supplementary Fig. S6). The fact that the intracellular phenotypes of *ben1* and *bex1* double mutant combinations with *are2* were much stronger than their seedling phenotypes is somewhat surprising and does provoke discussion.

### ALA3 together with BEN1 and BEX1 are involved in plant adaptation and recovery from osmotic stress

The defects in vesicular trafficking and abnormal organization of proteins at the PM, which we have observed in the *are* mutants, raised the question about their sensitivity to the osmotic stress conditions. Our previous research showed that exogenous application of hyperosmotic solutions enhanced the internalization of several tested PM proteins, including PIN2 (Zwiewka *et al*., 2015), which are known to be rapidly shuttling between the PM and endosomes (Geldner *et al.*, 2001).

**Fig. 5.**
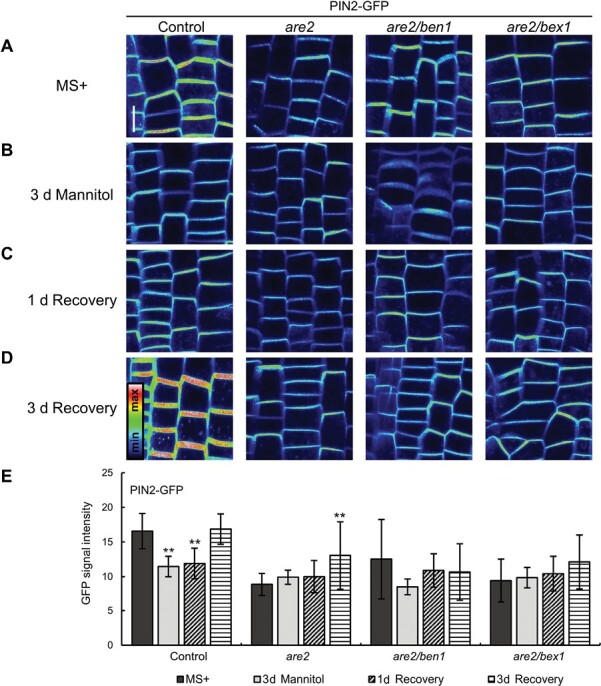
ALA3, BEN1, and BEX1 are important for the regulation of PM cargo delivery under osmotic stress conditions. (A–D) Maximal projection (*z*-stack=5 slices of 1 μm) of the epidermal root cells from PIN2–GFP and its crosses with *are2*, *ben1*, *bex1*, and the double mutants. Seedlings grown on MS+ medium were imaged (A), transferred to plates containing 200 mM mannitol, and grown for 3 d, after which images were taken again (B). To recover from stress, seedlings were transferred to fresh MS+ plates and imaged after 1 d (C) and 3 d (D). Scale bar=10 µm. (E) To quantify the results, the mean gray value was measured in selected single planes of each image. Error bars indicate the SD (12–15 seedlings per line at each time point). Asterisks indicate significant differences between initial MS+ and following stress/recovery treatments within each line (Student’s *t*-test, **P<0.01). The experiment was carried out three times with similar results.

To gain insight into how osmotic stress influences subcellular trafficking in *are* mutants, and to investigate whether ALA3, as well as BEN1 and BEX1 proteins, function as trafficking regulators in response to osmotic stress, we introduced the *are2/ben1* and *are2/bex1* double mutants into the PIN2–GFP genetic background. This rapidly routed efflux carrier is an excellent tool for visualizing subcellular trafficking alterations. Next, we measured the PIN2–GFP signal intensity on the PM upon hyperosmotic stress treatment and subsequent recovery. Initially, images of root epidermal cells of 5-day-old seedlings were taken (Fig. 5A). We were aware that the GFP signal in *are2* is lower already under the control conditions (Fig. 1C, D), and a similar decrease was observed in both *are2/ben1* and *are2/bex1* double mutants (Fig. 5A, E). Here, we wanted to investigate how such differences between the mutants and control would change during the stress treatment. Thus, the seedlings were transferred to plates containing MS+ supplemented with 200 mM mannitol and imaged after 3 d (Fig. 5B). While a significantly lower GFP signal was observed in the control line compared with the initial MS+ treatment, it remained unchanged in all tested mutant lines (Fig. 5B, E). To visualize recovery of PIN2 levels on the PM after osmotic stress, we transferred the seedlings back to plates containing MS+ and imaged the root cells after 1 d and 3 d. In the control, 1 d was not enough to observe the signal increase on the PM (Fig. 5C, E), but it was restored after 3 d of recovery (Fig. 5D, E). A different situation was observed in *are2*, where the PM PIN2 levels remained unchanged after the mannitol treatment and increased significantly only after 3 d of recovery, when even stronger signal intensity was measured compared wiht the initial MS+ measurements (Fig. 5E). For the double mutants, levels of PIN2 proteins on the PM remained unchanged after stress at both 1 d and 3 d of recovery (Fig. 5A–E). Similar results were obtained when analyzing the *are3* mutant allele (Supplementary Fig. S7). Compared with the dynamically changing signal levels in the control, less significant changes were observed in *are3*, and similarly in *are2.* The GFP signal intensity remained rather unchanged in both double mutants (Supplementary Fig. S7E).

**Fig. 6.**
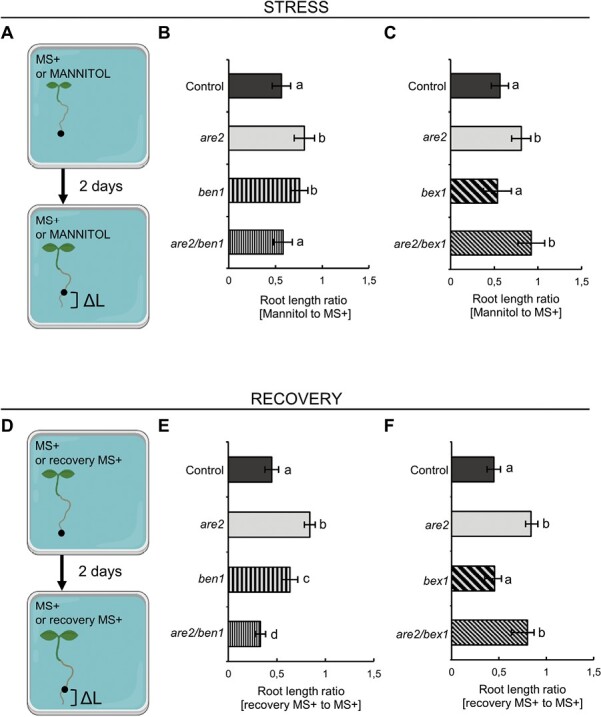
ALA3, BEN1, and BEX1 play a role in the root growth adaptation under osmotic stress conditions. (A–C) Five-day-old seedlings were transferred to MS+ plates with or without 200 mM mannitol. The root tips were marked, and seedlings were grown for 2 d (A). Root length after transfer (ΔL) was quantified in the control, *are2*, *ben1*, and *are2/ben1* (B) or *bex1* and *are2/bex1* (C) and expressed as the ratio of mannitol versus seedlings grown only on MS+ medium. Value 1 on the *y*-axis represents the average root length of seedlings grown only on MS+ plates. (D–F) Recovery from the osmotic stress was evaluated by transferring seedlings from (A–C) to fresh MS+ medium-containing plates, marking their root tips, and growing them for 2 d (D). ΔL was quantified in the control, *are2*, *ben1*, and *are2/ben1* (E) or *bex1* and *are2/bex1* (F) and normalized to seedlings grown only on MS+ medium. Value 1 on the *y*-axis represents the average root length of seedlings grown only on MS+ plates. Error bars indicate the SD, and average results for two independent experiments (15–20 seedlings) are presented. Columns sharing the same letters are not significantly different from each other (one-way ANOVA with Tukey post-hoc test, *P*<0.01).

Functional PIN trafficking ensures the directional transport of auxin, which is crucial for a plethora of developmental and growth processes, including the regulation of root elongation (Adamowski and Friml, 2015). The *ala3* mutants exhibit shorter primary roots compared with the wild-type seedlings (Poulsen *et al.*, 2008; Zhang *et al.*, 2020) (Supplementary Fig. S2A, B), and it was shown that the root growth varies with different temperatures and soil conditions (McDowell *et al.*, 2013). We aimed to determine whether osmotic stress influences root growth of *are* mutants and if the process of adaptation requires cooperation of ALA3 with ARF-GEFs and ARFs. Seedlings of *are2*, *ben1*, *bex1*, and their double mutants *are2/ben1* and *are2/bex1*, all in the PIN1–GFP background, were germinated together with the PIN1–GFP control on plates containing MS+ medium. After 5 d, they were transferred to fresh control plates with either MS+ or MS+ supplemented with 200 mM mannitol. The positions of root tips were marked, and seedlings were grown for another 2 d (Fig. 6A; Supplementary Fig. S8). Due to differences in the primary root length already at the start of the experiment in some of the mutants (shown also in Fig. 4E, F), the subsequent measurements of root growth after the transfer (ΔL) were normalized to control seedlings grown only on plates containing MS+. This enabled evaluation of the effect of mannitol treatment on root growth, correcting for the initial differences in root length between the analyzed mutant lines. In comparison with the control, root growth of *are2* seedlings was less affected by osmotic stress (Fig. 6B, C) and the same pattern was observed in the *ben1* mutant (Fig. 6B). Interestingly, we have observed a rescue of the phenotype in *are2/ben1* (Fig. 6B). In *bex1*, root growth was similar to the control in osmotic stress. However, *are2/bex1* seedlings responded in the same fashion as *are2* (Fig. 6C). To further investigate the root growth in response to osmotic changes, we analyzed its recovery. All seedlings from Fig. 6A–C were transferred to fresh MS+ plates. The seedlings that were previously subjected to mannitol are indicated as ‘recovery MS+’. We marked the root tips, measured ΔL after 2 d (Fig. 6D), and we calculated the ‘recovery MS+’/MS+ ratio. Compared with the control, an increase in root length was observed in *are2* and *ben1* mutants. On the other hand, we measured a major decrease in the *are2/ben1* double mutant (Fig. 6E). Response of root growth in *bex1* was identical to that of the control. The *are2* phenotype was even more pronounced in the *are2/bex1* double mutant, exhibiting a similar root growth response to the *are2* single mutant (Fig. 6F).

Our results indicate that loss of *ALA3* causes an impaired and less dynamic response to osmotic stress on both cellular (Fig. 5) and seedling levels (Fig. 6), which might be manifested as lack of the ability to quickly adapt the rate of PIN2 trafficking or the primary root growth during external conditions changes, and that together with BEN1 and BEX1, ALA3 plays an important role in plant adaptation to osmotic stress.

Additionally, we utilized RT–qPCR to examine the expression levels of *ALA3* in Col-0 seedlings treated with 200 mM mannitol for 3, 6, 9, 12, and 24 h (Supplementary Fig. S9). We detected a significantly increased *ALA3* expression after 9 h and 12 h of mannitol treatment, compared with mock treatment. We believe that this might further support the role of ALA3 in response to osmotic stress.

## Discussion

### ALA3 genetic lesion affects PIN plasma membrane distribution

The dynamic character of cell membranes and variable lipid composition between their two leaflets were described previously (Sanders and Mittendorf, 2011). Phospholipids are the major membrane components and are referred to as phosphatidylcholine (PC), phosphatidylethanolamine (PE), phosphatidylserine (PS), and phosphatidylinositol (PI), based on the head group that they contain. PC and PE are the most abundant classes of phospholipids in many organisms, including plants (Nakamura, 2017). Another class of structural lipids that we can find in cell membranes are sphingolipids with a ceramide backbone, and sterols, the main non-polar lipids. In *A. thaliana*, PS and PI are required for the PM recruitment and clustering of PIN1 and PIN2 in specialized sterol microdomains (Li *et al.*, 2021). PM clustering has been proposed to maintain the PIN polar distribution that enables the directional auxin transport between the cells (Kleine-Vehn *et al.*, 2011). Considering that flippases such as ALA3 translocate PS from the outer to the inner membrane leaflet (Poulsen *et al.*, 2008), they might play a role in maintenance of the asymmetric localization of PINs. Although detailed features and functions of PIN microdomains are still being characterized, they were mentioned as one of the mechanisms for limiting the PIN PM diffusion that can affect the PM abundance of those auxin efflux carriers (Kleine-Vehn *et al.*, 2011). In agreement with this, we observed a decreased PIN1–GFP and PIN2–GFP signal at the PM in *are2*, probably due to alterations in multiple processes, including decreased diffusion, polar exocytosis, and localized endocytosis, that strive to maintain the asymmetric PIN localization (Kleine-Vehn *et al.*, 2011). Consistent with the above, we measured a higher lateral diffusion rate of PIN2–GFP at the PM of *are*2 and *ala3-4*. It is likely that multiple cellular processes are affected by flipping lipids, especially PS, between the PM leaflets, which is connected to diffusion limitation and abundance of polar cargos, such as PIN2, in the PS-stabilized sterol microdomains. Certainly, as published before (Kleine-Vehn *et al.*, 2011), some *ala3*/*are* phenotypes will be PIN and auxin related, but not all of them, since the mutation might influence PM properties as we show later in this study. We could speculate that the absence of ALA3, or flippases in general, might alter lipid distribution between the two leaflets and affect the lateral diffusion-sensitive localization of PINs in the PM as prototypical polar cargos. However, more detailed and technically challenging investigation of the individual PM leaflets lipidic content would have to be done to resolve the above-discussed phenomena.

### ALA3 flippase participates in vesicle formation at the PM and TGN

Maintenance of lipid asymmetry between the two PM leaflets depends on multiple factors, including the ability of lipids to spontaneously cross the bilayer, the mechanisms of their retention in one membrane leaflet, and the functioning of transporters which actively facilitate their translocation (van Meer *et al*., 2008). One such group of transporters are the P4-ATPases (flippases), integral membrane pumps that flip phospholipids to the cytoplasmic membrane leaflet at the expense of ATP hydrolysis (Palmgren and Nissen, 2011). The flippase-mediated lipid relocation contributes to the formation of transport vesicles by generating an imbalance in lipid levels between the two leaflets, thus promoting the inwardly directed membrane deformation and subsequent curvature that is required for the vesicle budding (Graham, 2004; Takeda *et al*., 2014). In *A. thaliana*, P4-ATPases comprise 12 flippases named ALAs (Axelsen and Palmgren 2001). All ALAs characterized to date are strictly dependent on the association with a β-subunit, known as the ALA-interacting subunit (ALIS), to exit the endoplasmic reticulum and reach their final subcellular destination (López-Marqués *et al.*, 2010). Some of the P4-ATPases localize to multiple subcellular organelles. This also applies to ALA3, which localizes to the Golgi, TGN, PM, and endosomal membranes (Poulsen *et al.*, 2008; Zhang *et al.*, 2020). Therefore, we expected that the ALA3-associated defects might be detected on the membranous organelles within the cell on which the vesicles are most intensively formed, such as the PM and TGN. In agreement with this, we observed defects related to those two membranous domains in *are* mutants.

Firstly, we saw less PM staining by FM4-64 that might be connected to decreased intercalation of lipids by the dye. Our results support the observation of lower staining of the root epidermal cells in another *ala3* mutant that has been shown previously (Zhang *et al.*, 2020). A similar observation was made for *ala6/7* mutants, where the reduced ability of FM dye to stain the pollen tube PM was associated with changes in the PM’s surface charge or fluidity, which may be caused by altered lipid composition (McDowell *et al.*, 2015). Even though the pollen tube growth is impaired in *ala3*, the lipidomic analysis did not show changes in lipid composition (McDowell *et al.*, 2013). Instead, the defects were related to changes in local accumulation of the anionic PS at the cytoplasmic PM leaflet caused by a lack of ALA3 function (Zhou *et al.*, 2020; Yang *et al.*, 2022). Consistently, crosses of the *ala3* knockout mutant with plants harboring 2×mCherry-C2LACT (mCH-C2LACT) showed a decreased PM signal of this PS biosensor in the epidermal root cells, which was interpreted as an indicator of altered lipid composition (Zhang *et al.*, 2020). It is worth noting, as a word of caution, that expression levels of the sensor across the root were not investigated thoroughly to relate ratiometrically the signal intensity with the PS PM levels. To fully resolve those considerations, meticulous lipidomic studies of individual PM leaflets would be required.

Moreover, we detected an abnormal structure of the mutant TGN, which functions as a crucial vesicular hub in plant cells, where the cargo is sorted and subsequently delivered to the PM via the exocytic pathway. When we addressed secretion in *are* mutants seeds by visualizing the extruded seed coat mucilage, we observed a striking decrease in the thickness of the pectinous layer. Altogether, these results indicate that part of secretory pathway dependent on the function of the TGN, where fission and/or budding of the free secretory vesicles take place (Young *et al.*, 2008), is impaired in the *are* mutants. Similar observations were made in a previously published *ala3* mutant exhibiting aggregation of the TGN upon ARF1 immunolocalization and increased BFA body size (Zhang *et al.*, 2020). Lack of functional ALA3 can give rise to pleiotropic phenotypes, such as defective polar growth, plant development, lipid homeostasis, or adaptation to stress (Lopez-Marques, 2021), all of which may be related to membrane trafficking defects. Interestingly, *are* mutant genetic lesion did not affect the subcellular pattern of actin, which provides the tracks along which cytoplasmic vesicles are moved (DePina and Langford, 1999; Narasimhan *et al.*, 2020). The additional FRAP analysis that we performed did not detect any alterations in intracellular vesicle movement. Thus, we suggest that membrane functioning of ALA3 has a direct impact on the formation of transport vesicles and their budding-off from the Golgi/TGN or the PM, but it does not affect their cytoplasmic transit between these compartments. However, the process of vesicle formation does not depend only on the flippase activity, as it requires an interplay of more regulators. In yeast, the Neo1p and Drs2p flippases interact with ARFs, which are involved in generation of membrane curvature and recruitment of coat proteins in the early stages of vesicle budding, and GEFs, which function as the ARF activators (Wicky *et al*., 2004; Natarajan *et al.*, 2009). Additionally, it was proposed that interaction of Drs2p with Gea2p ARF-GEF stimulates the flippase activity, which in turn triggers recruitment of proteins needed for the vesicle formation to specific donor membrane locations (Natarajan *et al.*, 2009). ALA3 flippase is a plant ortholog of Drs2p and, analogously, it was shown to interact genetically and physically with the ARF-GEFs GNOM and BIG3 (Zhang *et al.*, 2020). These findings, together with trafficking defects localized in early endocytic pathways observed in the *bex1* mutant of the ARF1A1C/BEX1 (Tanaka *et al.*, 2014), the *ben1* mutant of the BIG5/BEN1 ARF (Tanaka *et al.*, 2009), and critically in the *are* mutants, led us to examine this trio in double mutant combinations. We addressed exocytosis/secretion and looked at seedling and grown plant phenotypes in the double mutants. The results that we obtained from all analyses had common features. We observed that the *are2* mutation masked the phenotypes of both *ben1* and *bex1*. Intriguingly, *are2/bex1* had more severe defects on both cellular and seedling/plant levels than *are2/ben1*. In *A. thaliana*, only one class of ARF GTPase, represented by the isoforms of ARF1, is required for all membrane trafficking processes, and this class can be activated by all ARF-GEFs that are functioning redundantly in distinct trafficking pathways (Singh *et al.*, 2018). Therefore, we speculate that the activity of BEN1 might be replaced by another ARF-GEF in *are2/ben1*, which could explain the less severe mutant phenotype. In contrast, mutation in ARF1, which plays an essential role in vesicle formation, can lead to amplified defects that we observed in *are2/bex1*.

### ALA3, BEN1, and BEX1 play a role in seedling adaptation to osmotic stress

Plants have developed multiple ways of responding to environmental changes, ranging from alterations in gene expression to morphological changes. For example, overexpression of the *AtRab7* vesicular trafficking regulator resulted in enhanced salt and osmotic stress resistance (Mazel *et al.*, 2004). Here, we observed that the expression of *ALA3* increased under osmotic stress. However, one of the most relevant mechanisms of adaptation to stress concerns the delivery of membrane material between the organelles mediated by a complex vesicular trafficking system (Levine, 2002). As shown also in this research, P4-ATPases participate in vesicle budding and, in addition, they have been connected to biotic and abiotic stress adaptation in diverse life forms. Cold-sensitive growth and trafficking defects were observed in the mutant of yeast Drs2p, the most extensively studied P4-ATPase which is required for protein transport from the TGN (Ripmaster *et al*., 1993; Chen *et al.*, 1999). Moreover, the double mutant strain of Drs2p with its interacting ARF-GEF Gea2p, *drs2Δgea2Δ*, shows even stronger cold-sensitive phenotype than the single *drs2Δ* (Chantalat *et al.*, 2004). Analogously, plant ALA3 flippase interacts with ARF-GEFs GNOM and BIG3 (Zhang *et al.*, 2020) and, interestingly, GNOM has been shown to mediate cold stress response in plants (Ashraf and Rahman, 2019). While both *ala3* (McDowell *et al.*, 2013) and *gnom* (Ashraf and Rahman, 2019) seedlings exhibit cold-sensitive phenotypes, the temperature sensitivity of their double mutant remains unknown. The double mutant combinations of ARF and ARF-GEF with the flippase, that we examined here, to some extent fill this knowledge gap; however, rather than during a temperature challenge, we tested them under osmotic stress conditions. It has been demonstrated that plant cells rapidly adjust rate between endocytosis and exocytosis in response to changes in turgor pressure (Nakayama *et al.*, 2012; Zwiewka *et al*., 2015). However, to date, there has been no evidence of flippases or ARF-GEFs being involved in adaptation to osmotic stress. BEN1 is however required for triggering the responses to biotic stress (Gangadharan *et al.*, 2013) and its activity is important for PIN2 recycling during adaptation to oxidative stress (Zwiewka *et al*., 2019). Importantly, the role of ARF1 in osmotic stress defense has been reported, showing that overexpression of *ARF1* from *Spartina alterniflora* (*SaARF1*) results in salt and drought tolerance and improved cell membrane integrity in rice and Arabidopsis transgenic plants (Joshi *et al.*, 2014; Karan and Subudhi, 2014). We mimicked the drought conditions utilizing mannitol while PIN2 PM abundance served us to monitor changes in vesicular trafficking. In our hands, mannitol treatment resulted in depletion of PIN2 from the PM in the control, which is consistent with previously published data showing the enhancement of endocytosis after osmotic shock (Zwiewka *et al*., 2015; Couchoud *et al.*, 2019). After transferring the seedlings back to normal media, the PM PIN2 levels were re-established in the control. For *are* and *ben1*, as well as *bex1* double mutant combinations, we did not observe such PM PIN2 fluctuation. This made us speculate that impaired function of the flippase, ARF-GEF, or ARF not only affected the process of vesicle formation mentioned above but ultimately decreased the ability of plant cells to quickly adjust intracellular trafficking rates during adaptation to osmotic stress. Mannitol treatment was also shown to inhibit growth of seedling primary roots (Cajero-Sanchez *et al.*, 2019). The *ala3/are* seedlings exhibit a short root phenotype already in standard growth conditions (Poulsen *et al.*, 2008; Zhang *et al.*, 2020) (Supplementary Fig. S2A, B), but when we corrected for that by measuring their growth rate change, we showed that they are resistant to mannitol. Whereas root length in *are2/ben1* was reminiscent of the control, we observed a strong resistance to mannitol in the *are2/bex1* double mutant. Note that by the resistance we mean lack of the normally occurring root growth inhibition in plant response to the osmotic stress. The outline above suggests that some of the ARF-GEFs might function redundantly in part of the subcellular trafficking pathways, thus taking over the function of BEN1, resulting in milder phenotypic defects of *are2/ben1*. In contrast, the function of ARF1 is essential for multiple cellular processes ensuring the plant’s vitality, and excluding both ARF1 and ALA3 from the vesicle formation process leads to severe defects observed in *are2/bex1*. Altogether, we have shown the importance of ALA3 flippase in facilitating dynamic changes of subcellular trafficking rates that modulate the delivery of cargos, including PIN auxin efflux carriers, and lipids to the PM. Those steps in turn enable adjustment of root growth during plant adaptation to osmotic stress. We have shown this when using mannitol to simulate the water deficits, but it is known that the roots continually adjust their growth rate when penetrating the soil layers that can have varying water availability and osmotic potential (Deak and Malamy, 2005; Munns and Tester, 2008; Galvan-Ampudia and Testerink, 2011; Cajero-Sanchez *et al.*, 2019). Our data hint that the delivery of vesicles and cargo to the PM is changing dynamically in different osmotic conditions, which might be relevant for PM integrity preservation that we have hypothesized previously (Zwiewka *et al*., 2015). In conclusion, we show that dynamic changes of endo- and exocytosis are more important in aspects of plant homeostasis maintenance than has been thought, with their role reaching beyond the PIN trafficking in root gravitropism and root elongation control (Abas *et al.*, 2006; Kleine-Vehn and Friml, 2008; Kleine-Vehn *et al*., 2010).

## Supplementary data

The following supplementary data are available at *JXB* online.

Fig. S1. FRAP of PIN2–GFP in *ala3* mutants.

Fig. S2. Auxin-related phenotypes of the *are* mutants.

Fig. S3. Optimization of FM4-64 staining.

Fig. S4. Additional FRAP analyses of endosomal movement dynamics.

Fig. S5. ALA3 is necessary for proper morphology of the TGN compartments.

Fig. S6. Morphology of 38-day-old plants.

Fig. S7. ALA3, BEN1, and BEX1 are important for the regulation of cargo delivery under osmotic stress conditions.

Fig. S8. Seedling phenotype 2 d after transfer to fresh plates.

Fig. S9. RT–qPCR analysis of *ALA3* expression in response to osmotic stress.

erad234_suppl_supplementary_figures_S1-S9

## Data Availability

All data supporting the findings of this study are available within the paper and within its supplementary data published online.

## Abbreviations

*are*: *auxin resistant endocytosis*
ARF: ADP ribosylation factor
*ben1*: *BFA-visualized endocytic trafficking defective1*
*bex1*: *BFA-visualized exocytic trafficking defective 1*
BFA: brefeldin A
CFP: cyan fluorescent protein
EE: early endosome
FRAP: fluorescence recovery after photobleaching
GAP: GTPase-activating protein
GEF: guanine nucleotide exchange factor
GFP: green fluorescent protein
PC: phosphatidylcholine
PE: phosphatidylethanolamine
PI: phosphatidylinositol
PIN: PIN-FORMED
PM: plasma membrane
PS: phosphatidylserine
ROI: region of interest
TGN: *trans*-Golgi network
