# Supplementary material for: Arabidopsis flippase ALA3 is required for adjustment of early subcellular trafficking in plant response to osmotic stress
Source: J Exp Bot. 2023 Jun 23;74(17):4959–77. doi: 10.1093/jxb/erad234 (PMC10498020; doi:10.1093/jxb/erad234)
Supplement: erad234_suppl_supplementary_figures_S1-S9 [file erad234_suppl_supplementary_figures_s1-s9.pdf]

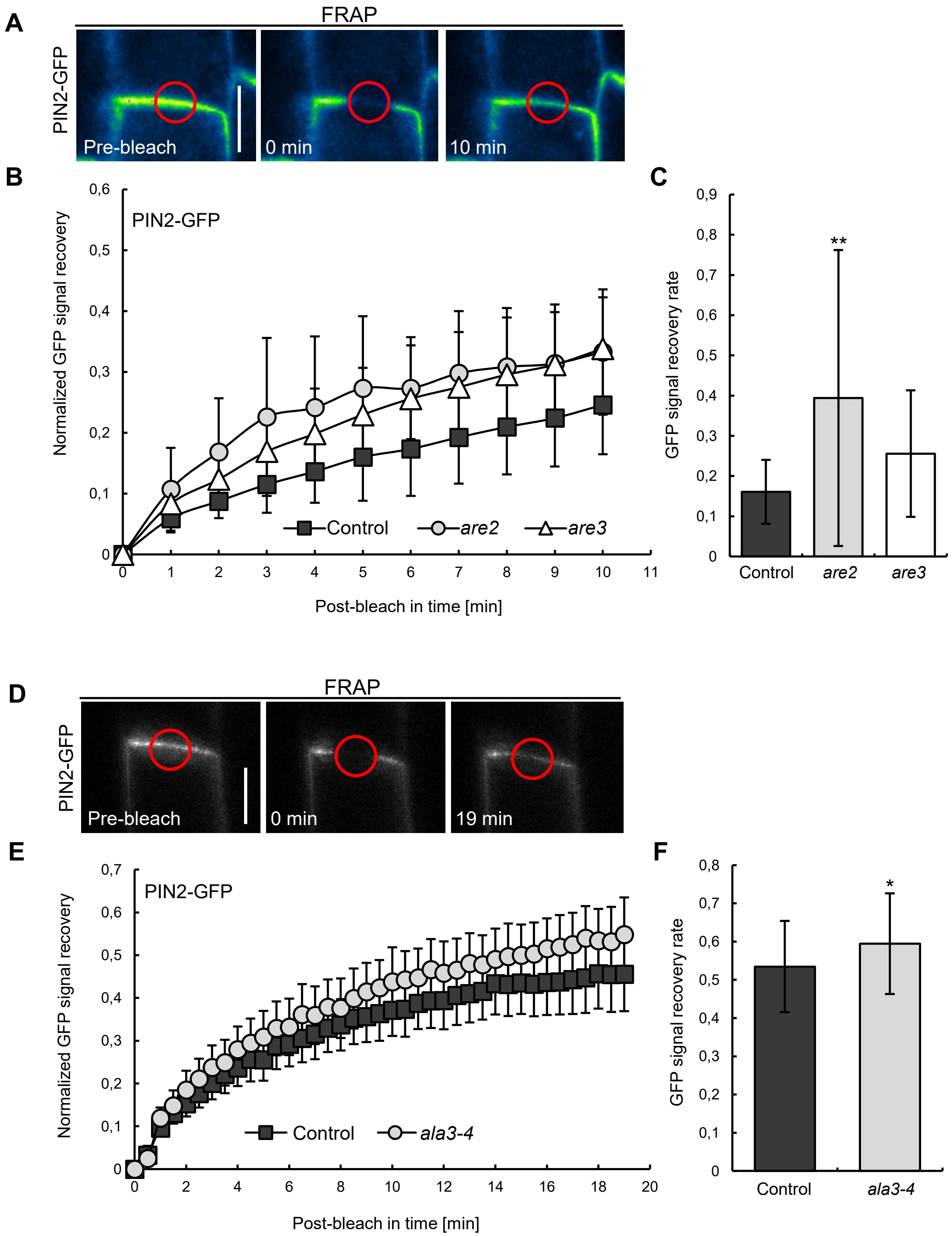
A
FRAP
PIN2-GFP
Pre-bleach
0 min
10 min
B
PIN2-GFP
Normalized GFP signal recovery
Post-bleach in time [min]
Control
are2
are3
C
GFP signal recovery rate
**
Control
are2
are3
D
FRAP
PIN2-GFP
Pre-bleach
0 min
19 min
E
PIN2-GFP
Normalized GFP signal recovery
Post-bleach in time [min]
Control
ala3-4
F
GFP signal recovery rate
*
Control
ala3-4

Supplementary Fig. S1 - FRAP of PIN2-GFP in *ala3* mutants

(A, D) Representative images of the PIN2-GFP marker in root epidermis acquired in the indicated timepoints. **(B)** Representative normalized recovery curve after FRAP analysis of PIN2-GFP control (squares), and its crosses with *are2* (circles) and *are3* (triangles). **(C)** Mean recovery rate expressed as percentage of GFP signal in PIN2-GFP and its crosses with *are2* and *are3* from 40, 43, and 40 individual FRAP experiments, respectively. Error bars indicate SD and asterisks mark significant differences (Student's t-test, **$P<0.01$). **(E)** Representative normalized recovery curve after FRAP analysis of PIN2-GFP in Col-0 (squares) and its cross with *ala3-4* (circles). **(F)** Mean recovery rate expressed as percentage of GFP signal in PIN2-GFP, and its cross with *ala3-4* from 40 individual FRAP experiments. Error bars indicate SD and asterisk marks significant difference (Student's t-test, *$P<0.05$).

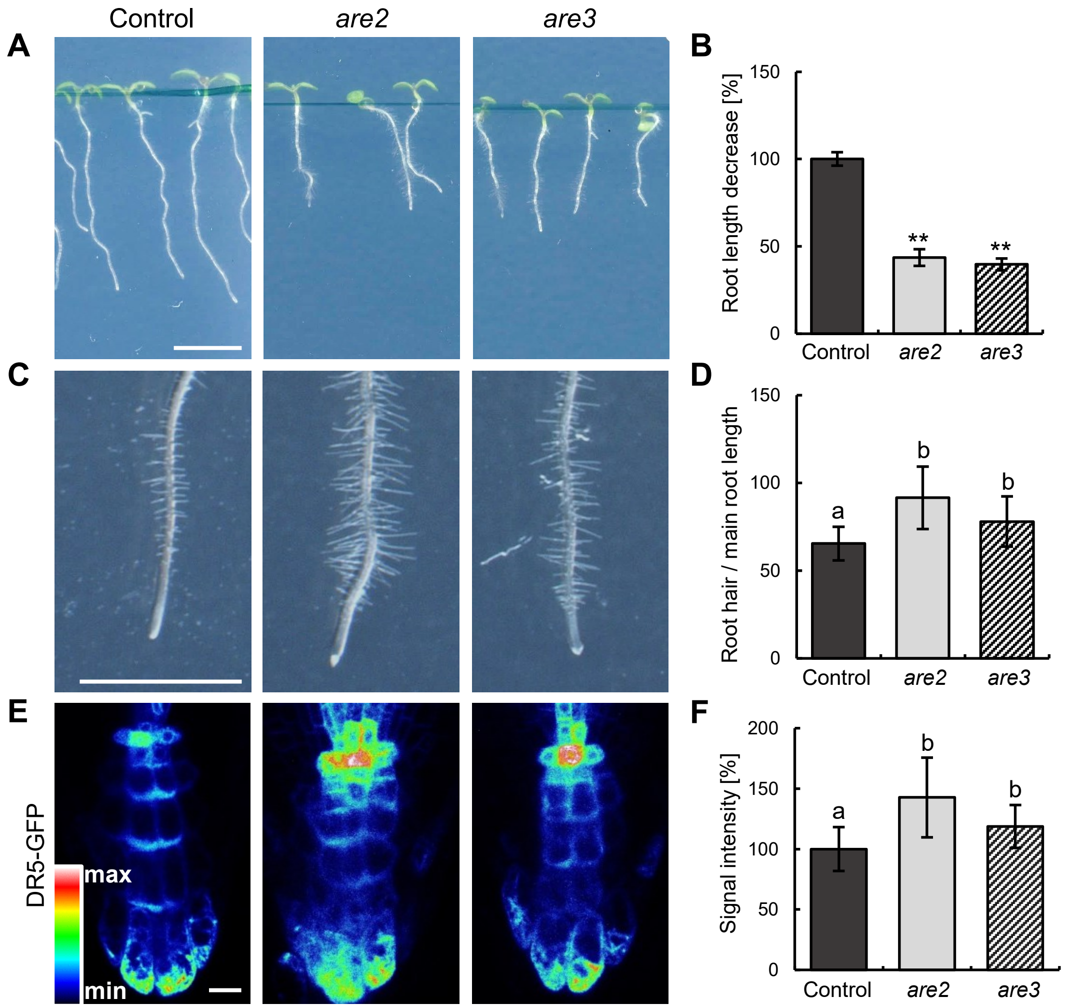

Supplementary Fig. S2 – Auxin-related phenotypes of the *are* mutants

(A) Seedling phenotype of the control, *are2* and *are3*, all in the PIN1-GFP genetic background. Scale bar = 5 mm. **(B)** Root length quantification. Error bars indicate SD (>20 seedlings). Asterisks mark significant differences (Student´s t-test, **P<0.01). **(C)** Magnification of the control, *are2* and *are3* roots. Scale bar = 500 µm. **(D)** Quantification of the root hair number / main root length ratio. Error bars indicate SD (>20 seedlings). **(E)** Representative images of root tips expressing DR5-GFP shown the in pseudo-colour signal intensity scale. Scale bar = 10 µm. **(F)** Quantification of the DR5-GFP fluorescent signal in root tips. Error bars indicate SD (>20 seedlings). The columns in **D** and **F** sharing the same letters are not significantly different from each other (One-Way Anova with Tuckey post-hoc test, P<0.01).

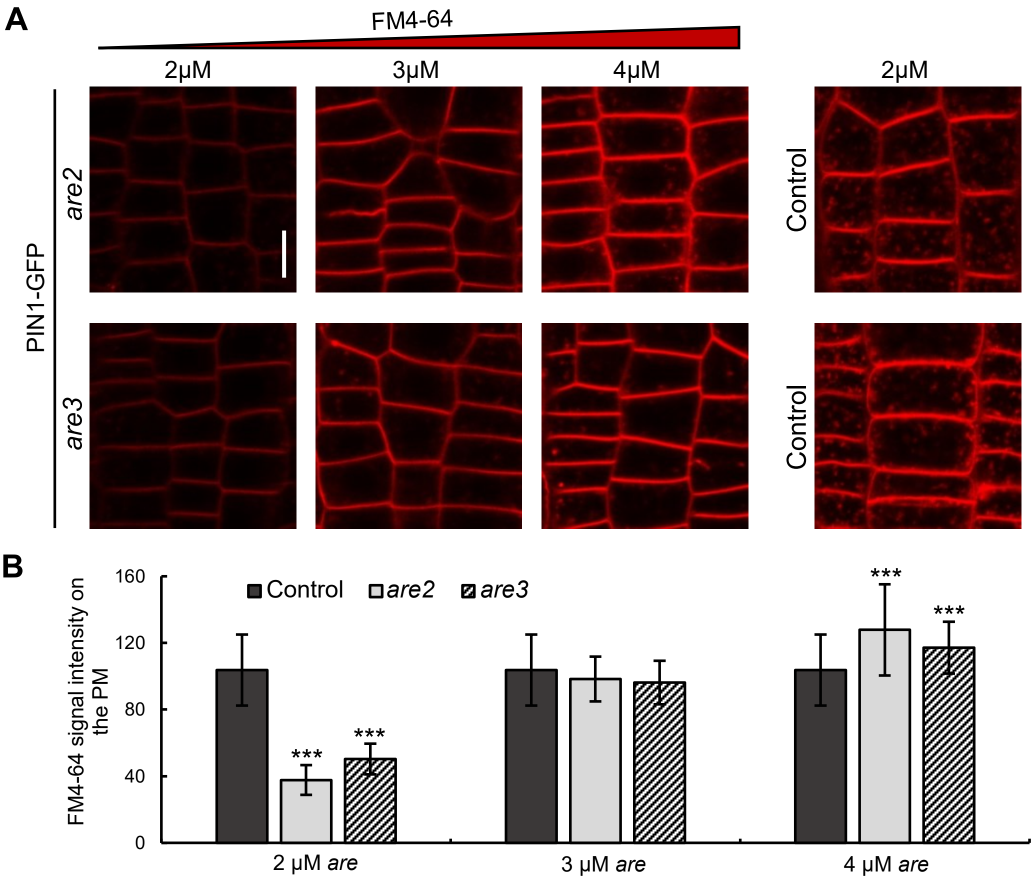

Supplementary Fig. S3 – Optimalization of FM4-64 staining

(A) To state the optimal concentration of FM4-64 which labels PM of the control and both mutants (all in PIN1-GFP genetic background) equally, the control seedlings were stained with 2 μM dye (panels on the right) and seedlings from *are2* and *are3* with (from the left side) 2 μM, 3 μM and 4 μM dye. Scale bar = 10 μm. **(B)** Quantification of the FM4-64 PM signal. Error bars indicate SD (35 cells). Asterisks mark significant differences (Student´s t-test, ***P<0.001).

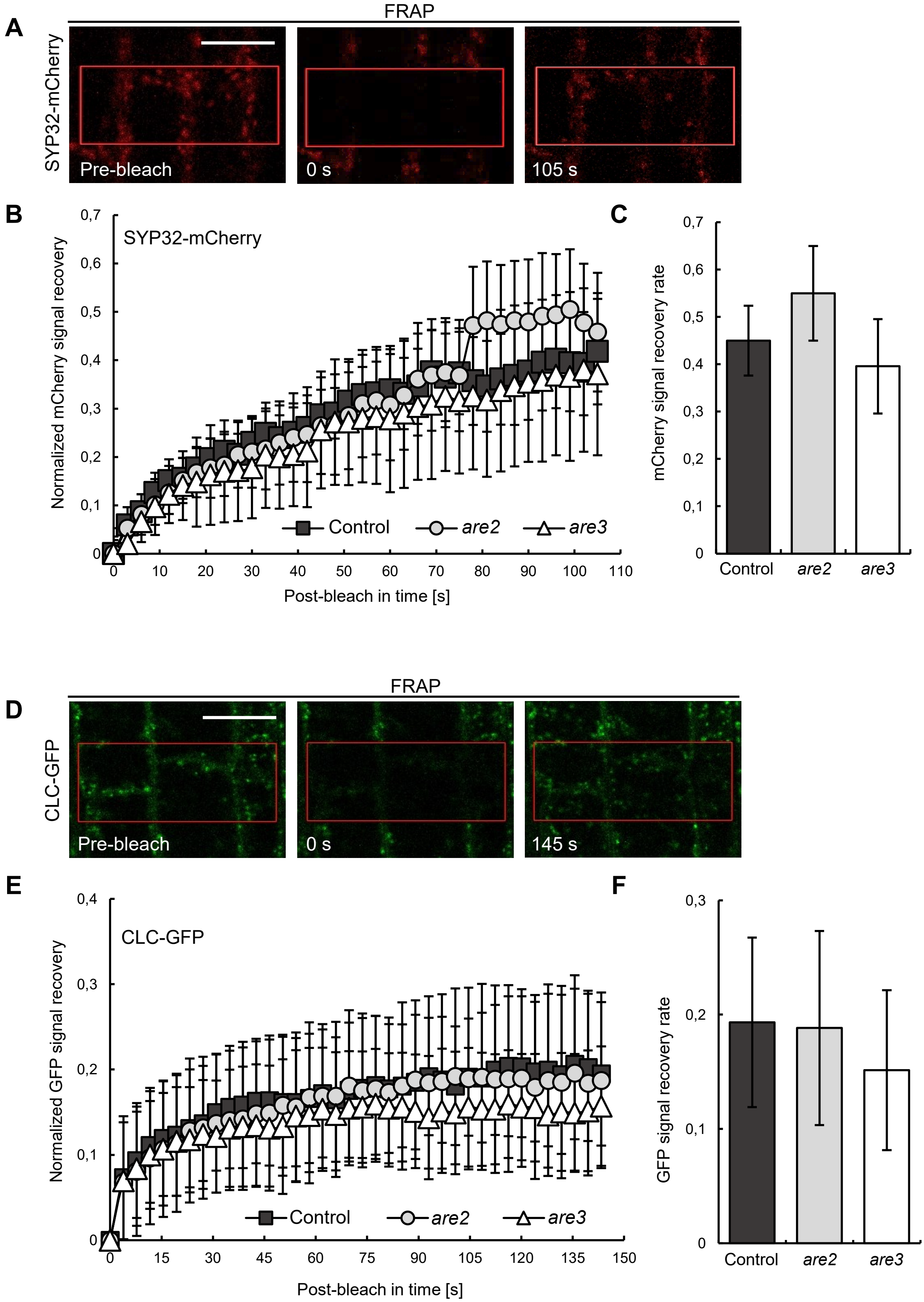
A
FRAP
SYP32-mCherry
Pre-bleach
0 s
105 s
B
SYP32-mCherry
Normalized mCherry signal recovery
Control
are2
are3
Post-bleach in time [s]
C
mCherry signal recovery rate
Control
are2
are3
D
FRAP
CLC-GFP
Pre-bleach
0 s
145 s
E
CLC-GFP
Normalized GFP signal recovery
Control
are2
are3
Post-bleach in time [s]
F
GFP signal recovery rate
Control
are2
are3

Supplementary Fig. S4 – Additional FRAP analyses of vesicular trafficking

(A) Representative images of the SYP32-mCherry marker in root epidermis acquired in the indicated timepoints of the FRAP experiment. **(B)** Representative normalized recovery curve after FRAP analysis of SYP32-mCherry in Col-0 (squares) and the mutant background crosses with *are2* (circles) and *are3* (triangles). **(C)** Mean recovery rate expressed as percentage of mCherry signal in SYP32-mCherry, and its crosses with *are2* and *are3* from 26, 27 and 29 individual FRAP experiments, respectively. **(D)** Representative images of the CLC-GFP marker in root epidermis acquired in the indicated timepoints of the FRAP experiment. **(E)** Representative normalized recovery curve after FRAP analysis of CLC-GFP in Col-0 (dark grey circles) and the mutant background crosses with *are2* (light grey circles) and *are3* (white circles). **(F)** Mean recovery rate expressed as percentage of GFP signal in CLC-GFP, and its crosses with *are2* and *are3* from 30 individual FRAP experiments. Scale bars = 20 μm.

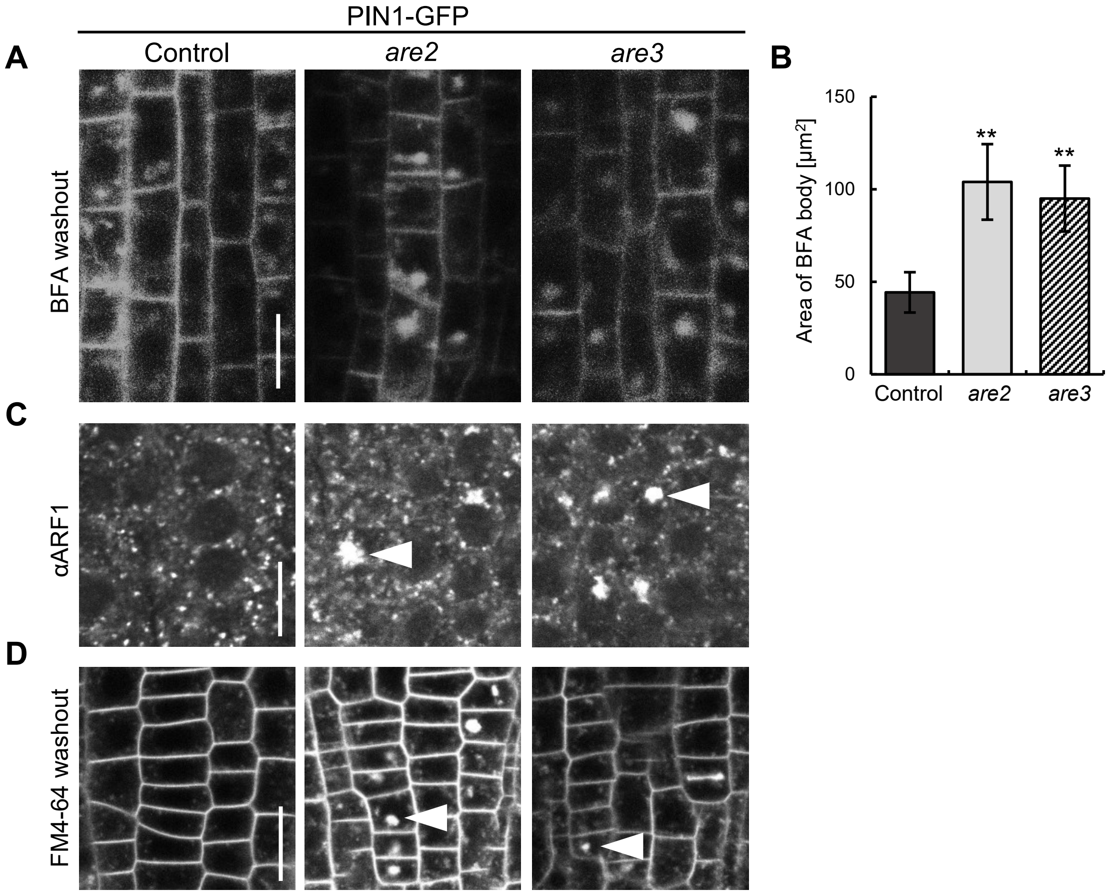

Supplementary Fig. S5 - ALA3 is necessary for proper morphology of the TGN compartments

(A) Visualization of PIN1 accumulation in BFA bodies after 1 h treatment followed by a 30 min MS+ washout for the control, *are2* and *are3*, all in the PIN1-GFP genetic background. Scale bar = 10 µm. **(B)** Area quantification of the PIN1-containing BFA bodies. Error bars indicate SD (>30 BFA bodies). Asterisks mark significant differences between single and double mutants (Student´s t-test, **P<0.01). **(C)** ARF1 immunolocalization in the root cells. White arrows indicate aggregations of ARF1 in TGN. Scale bar = 10 µm. **(D)** Epidermal root cells of seedlings stained with the FM4-64 lipophilic dye for 20 min followed by a 30 min washout. White arrows indicate intracellular aggregations. Scale bar = 10 µm.

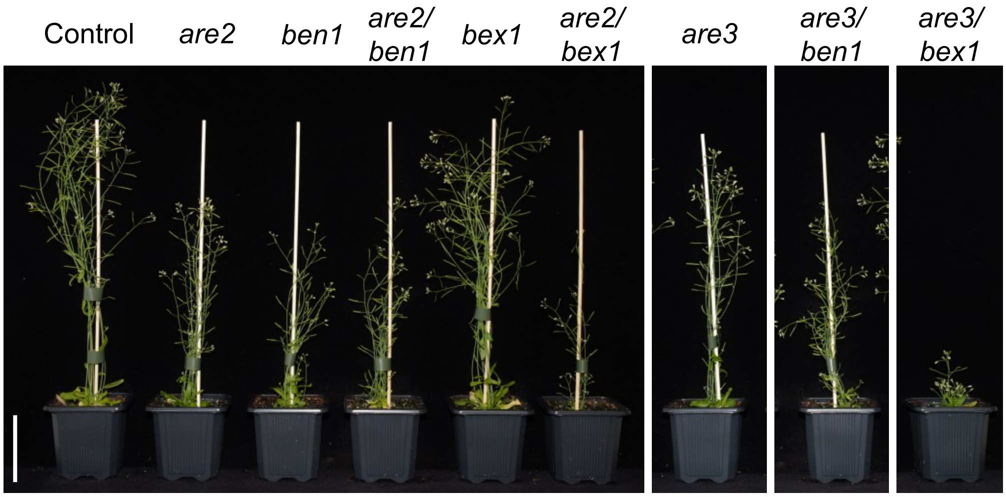

Supplementary Fig. S6 - Morphology of 38-day-old plants

Scale bar = 5 cm.

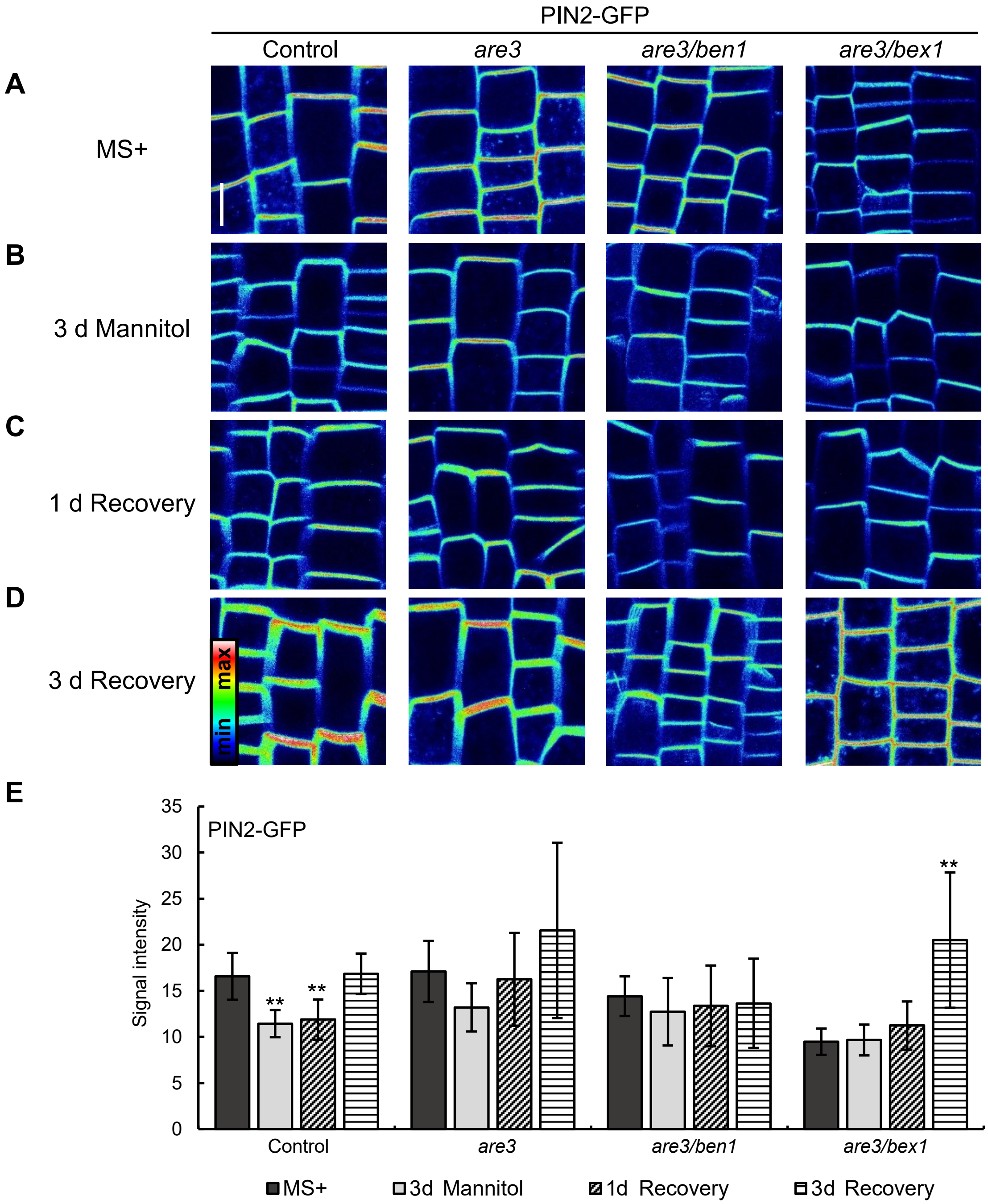
PIN2-GFP
Control
are3
are3/ben1
are3/bex1
A
MS+
B
3 d Mannitol
C
1 d Recovery
D
3 d Recovery
max
min
E
PIN2-GFP
Signal intensity
35
30
25
20
15
10
5
0
**
**
**
Control
are3
are3/ben1
are3/bex1
MS+
3d Mannitol
1d Recovery
3d Recovery

Supplementary Fig. S7 - ALA3, BEN1 and BEX1 are important for the regulation of cargo delivery under osmotic stress conditions.

(A-D) Maximal projection (z-stack, 5 slices of 1 µm) of the epidermal root cells from the control and its crosses with *are3*, *ben1*, *bex1* and the double mutants, all in the PIN2-GFP genetic background. Seedlings grown on MS+ medium were imaged **(A)**, transferred to plates containing 200 mM mannitol and grown for 3 days. Images were taken **(B)**. To recover from stress, seedlings were transferred back to the MS+ plates and imaged after 1 day **(C)** and 3 days **(D)**. Scale bar = 10 µm. **(E)** Mean gray value was measured in selected single planes of each image. Error bars indicate SD (12-15 seedlings per line in each time point). Asterisks indicate significant differences between initial MS+ and following stress/recovery treatments within each genotype (Student´s test, **P<0.01). The experiment was carried out three times with similar results.

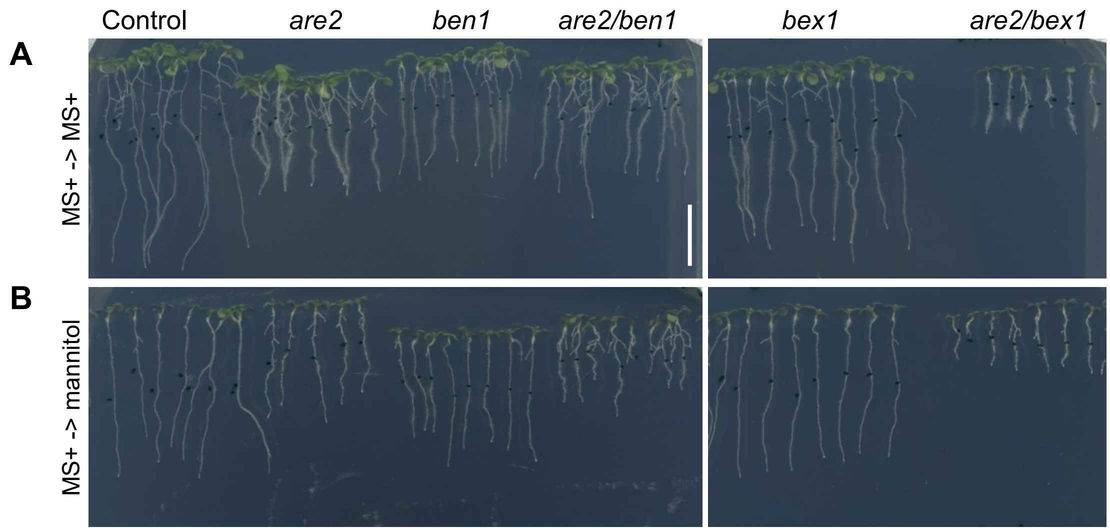

Supplementary Fig. S8 – Seedling phenotype 2 days after transfer to fresh plates

(A) Phenotype of seedlings grown on MS+ for 5 days, transferred to a fresh MS+ plate, grown for 2 days and scanned. **(B)** Phenotype of seedlings grown on MS+ for 5 days, transferred to a plate containing MS+ supplemented with 200 mM mannitol, grown for 2 days and scanned. Scale bar = 1 cm.

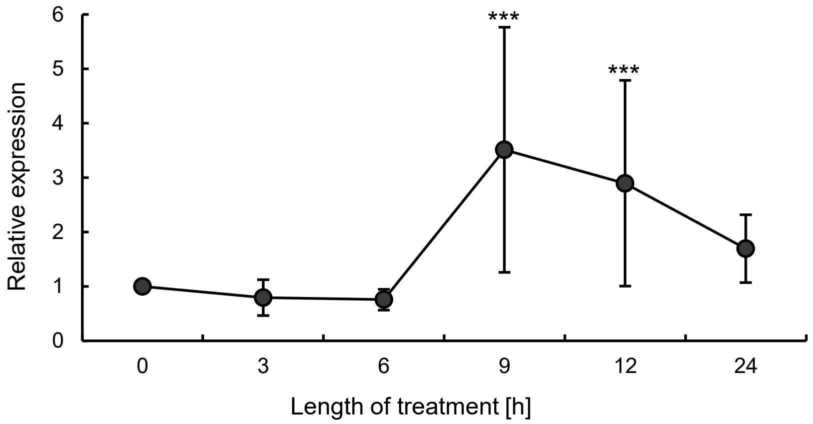

Supplementary Fig. S9 – RT-qPCR analysis of *ALA3* expression in response to osmotic stress

5-day-old Col-0 seedlings were transferred to liquid MS+ medium with or without 200 mM mannitol and then the plants were harvested at the indicated times. Data are shown as relative ratio of treated and non-treated samples in each time point (expression normalized to UBC10 and EF1alpha); mean +-SD of three independent experiments. Asterisks indicate significant differences between the mock treatment and mannitol treatment in given time points (***P<0.001)
